# Cytoglobin affects tumorigenesis and the expression of ulcerative colitis-associated genes under chemically induced colitis in mice

**DOI:** 10.1038/s41598-018-24728-x

**Published:** 2018-05-02

**Authors:** Mohammad Yassin, Hannelouise Kissow, Ben Vainer, Philomeena Daphne Joseph, Anders Hay-Schmidt, Jørgen Olsen, Anders Elm Pedersen

**Affiliations:** 1 Department of Cellular and Molecular Medicine, Faculty of Health and Medical Sciences, University of Copenhagen, Copenhagen, Denmark; 2 Department of Biomedical Sciences and NNF Center of Basic Metabolic Research, Faculty of Health and Medical Sciences, University of Copenhagen, Copenhagen, Denmark; 3 Department of Pathology, Rigshospitalet, University of Copenhagen, Copenhagen, Denmark; 4 Department of Neuroscience and Pharmacology, Faculty of Health and Medical Sciences, University of Copenhagen, Copenhagen, Denmark; 5 Department of Odontology, Faculty of Health and Medical Sciences, University of Copenhagen, Copenhagen, Denmark

## Abstract

Cytoglobin (Cygb) is a member of the hemoglobin family and is thought to protect against cellular hypoxia and oxidative stress. These functions may be particularly important in inflammation-induced cancer, e.g., in patients with ulcerative colitis (UC). In this study, we investigated the development of inflammation and tumors in a murine model of inflammation-induced colorectal cancer using a combined treatment of azoxymethane and dextran sulfate sodium. A bioinformatics analysis of genome-wide expression data revealed increased colonic inflammation at the molecular level accompanied by enhanced macroscopic tumor development in Cygb-deficient mice. Moreover, the expression of the UC-associated gene neurexophilin and PC-esterase domain family member 4 (Nxpe4) depended on the presence of Cygb in the inflamed colonic mucosa. Compared to wild type mice, RT-qPCR confirmed a 14-fold (p = 0.0003) decrease in Nxpe4 expression in the inflamed colonic mucosa from Cygb-deficient mice. An analysis of Cygb protein expression suggested that Cygb is expressed in fibroblast-like cells surrounding the colonic crypts. Histological examinations of early induced lesions suggested that the effect of Cygb is primarily at the level of tumor promotion. In conclusion, in this model, Cygb primarily seemed to inhibit the development of established microadenomas.

## Introduction

Cytoglobin (Cygb) is a heme-containing protein belonging to the hemoglobin family. The primary sequence of Cygb is approximately 40 amino acids longer than the primary structure of the other globins, and based on amino acid sequence comparisons, Cygb is more closely related to hemoglobin and myoglobin than to neuroglobin. In Cygb, the heme iron is hexacoordinated via an arrangement involving the imidazole groups of a proximal and a distal histidine^1^. Upon the binding of an exogenous ligand (e.g., O_2_, NO or CO), the distal imidazole group is displaced^1^. Given the ability to bind O_2_ and NO, Cygb has been suggested to be capable of counteracting both cellular hypoxia and oxidative stress (reviewed in^2^). Indeed, in a study of tetrachloride-induced liver fibrosis in rats, the subcutaneous administration of recombinant Cygb inhibited fibrosis development and increased the expression of liver proteins involved in the oxidative stress response^3^. Additionally, a recent study in rats reported increased Cygb gene and protein expression levels in the brain, heart and lungs after exposure to cigarette smoke^4^. Cygb up-regulation was also evident in tissue samples from patients with Barrett’s metaplasia, which is an early stage in the Barrett’s esophagus disease sequence that exhibits increased oxidative stress and mitochondrial instability compared with other stages of the disease^5^. Moreover, the activation of oxidative stress pathways in mice lacking functional Cygb gene expression (Cygb^−/−^ mice) was accompanied by tumor development in a model of steatohepatitis-induced hepatocellular cancer^6^. Interestingly, a long-term study of untreated Cygb^−/−^ mice demonstrated multiple organ abnormalities, including tumor development, paralleled by an increase in the urinary concentration of NO metabolites^7^. Therefore, the present knowledge of Cygb suggests that it plays an important protective role against oxidative stress, both in the long- and short-term aspects of inflammatory conditions, such as those underlying the development of fatty liver disease. With respect to cancer development, Cygb is best characterized as a tumor suppressor gene because Cygb appears to protect against cancer development in the liver and because Cygb expression is decreased in several human cancer types^2^. However, Cygb expression in cancer remains a matter of debate, and more studies are required to fully understand its roles.

Chronic inflammatory bowel disease (IBD), ulcerative colitis (UC) and Crohn’s disease (CD) are associated with an increased risk for the development of colorectal cancer (CRC) known as colitis-associated colorectal cancer (CAC). Moreover, inflammation-induced oxidative stress is believed to play an important role in the development of CAC (reviewed in^8,9^). Given the ubiquitous expression of Cygb, it is reasonable to speculate that Cygb plays a protective role against CAC development, and it is the purpose of the present work to investigate this hypothesis further. Toward this end, this study leveraged mouse genomics in combination with the azoxymethane (AOM)/dextran sulfate sodium (DSS) murine model of chemically induced CAC.

Thus, using the AOM/DSS model of inflammation-induced CRC, we investigated the colonic inflammation and tumor development in Cygb^−/−^ mice. Furthermore, we described both normal mouse gastrointestinal tract Cygb expression as well as its expression during chronic colitis.

## Materials and Methods

### Establishment of Cygb-deficient mice

A Cygb knockout (KO) mouse model was created by genOway (Lyon, France) under the project number genOway/JCO/HSA2-Cygb/010611. The mouse Cygb gene is located on chromosome 11, extends over 8.7 kb, and contains 4 exons, start and stop codons in exons 1 and 4, respectively. Two isoforms coding for the same protein of 190 amino acids have been described.

In contrast with Thuy *et al*.^6^, we chose to develop a Cygb conditional KO mouse model via the deletion of exon 2, which codes for the two heme-binding sites, resulting in a frame shift. A targeting vector was constructed with a long homology arm of 4.9 kb upstream of exon 2 and a short homology arm of 2.4 kb downstream of exon 2 for the 129 Sv mouse strain; two loxP511 sites were flanking exon 2. Two selection markers were inserted in the targeting vector; a positive neomycin gene flanked by FRT sites just downstream of the 3′ loxP511 site, and the negative selection marker diphtheria toxin A (DTA) outside of the homologous region of the short homology arm at the distal 5′ end of the targeting vector (Supplementary Figure S1).

The targeting vector was electrophoretically transferred into 129 Sv/Pas embryonic stem cells (ESCs), and G418-resistant ESCs were selected. A total of 407 clones were selected and amplified, and an initial PCR screening revealed 19 positive clones. Of these, 6 were verified by Southern blot, introduced into C57BL/6 J mouse blastocysts, transferred into OF1 pseudo-pregnant females, and carried to term. Chimeric mice were selected by coat color. For the *in vivo* deletion of the loxP-flanked region, highly chimeric males (more than 50%) were mated with C57Bl/6 J Cre-recombinase-expressing deleter females to allow the germ-line excision of the loxP-flanked exon 2, thereby generating the constitutive KO allele. Six heterozygous constitutive KO F1 progeny were obtained, as revealed by both PCR screening and Southern analyses. These were backcrossed with C57BL/6 J mice for 7 generations before being used for the experiments.

To obtain a sufficient number of mice for the experiment, the following breeding was conducted. Two pairs (a and b) of heterozygous mice were bred (F1xF1 breeding), and wild type (WT) and KO F2 offspring were selected. From these, a WT F2axF2b line and a KO F2axF2b line were established, and WT and KO F3 offspring were obtained. This method ensures the littermate character of the mice, but using far less mice. The Cygb^−/−^ mice did not show any signs of Cygb immunoreactivity (Cygb-ir) in the liver (Supplementary Figure S2) or in any other tissue examined (i.e., intestine, kidney, testis, heart, vessels (data not shown)). In addition, a genome-wide expression analysis using mouse gene ST arrays demonstrated a 6.5-fold decrease in Cygb expression in the colons of Cygb^−/−^ mice (Supplementary Table S1).

In this Cygb^−/−^ mouse model, fecundity was normal compared to the B6 background WT mice; equal rates of male and female offspring were observed, and no differences in overall health were observed between the WT and Cygb^−/−^ mice. In contrast to previous studies^7^, the occurrence of spontaneous cancer was not observed to differ between the WT and Cygb^−/−^ mice, even at old age. All mice were housed in a pathogen-free animal facility (Panum Instituttet, University of Copenhagen) with access to water and a standard rodent diet ad libitum. Mice were maintained under controlled conditions of temperature (22 ± 2 °C) and humidity (50 ± 10%) and a 12-hour light/dark cycle. The experimental protocols were approved by the Danish Veterinary and Food Administration, Ministry of Environment and Food of Denmark and performed according to Danish guidelines.

### Human biopsies

Human colon samples were obtained from the biopsies of 3 patients undergoing a partial colon removal due to colon cancer. The biopsies investigated from each patient contained non-cancerous segments. The biopsies were obtained from an existing biorepository and were used in an anonymous manner.

### Intestinal colonic crypt isolation and culture conditions

Intestinal crypt isolation was performed as previously described by Tan *et al*.^10^, with few modifications. In brief, the distal half of the colon from C57BL/6 WT mice, excluding the cecum and the most proximal portion, was harvested and transferred to a petri dish on ice; then, the sample was flushed with ice-cold phosphate-buffered saline (PBS) and cut open longitudinally. The open colon was washed again with ice-cold PBS with the intestinal lumen facing upwards and cut into 2 mm segments before being transferred into a 50 ml conical tube with 15 ml of ice-cold PBS. The small intestine sections were incubated with chelation buffer (PBS with ethylenediaminetetraacetic acid 3 mM and dithiothreitol 0.05 mM) for 60 min at room temperature and then suspended in 2 ml of cold PBS + 0.1% bovine serum albumin. Crypts were then detached from the basal membrane via moderate shaking to preserve the intact crypt structures. The colon segments were allowed to settle by gravity, and the supernatant was collected in a new 15 ml tube. This step was repeated 5–6 times, and the fractions with the most enriched, intact crypts with the fewest single

cells were used for further culturing. The crypts from the chosen fractions were then centrifuged at 200 × g for 5 min to remove single cells and to obtain a pellet with pure crypts. The pellet was resuspended in 10 ml of cold Dulbecco’s modified Eagle’s medium/F12 (Life Technologies/Thermo Fisher Scientific, MA, USA), and the crypts were counted and assessed using an inverted microscope.

The culturing step was conducted according to section 2 in a protocol from STEMCELL Technologies Inc.; crypt cells were pelleted and resuspended in 150 µl of Matrigel Matrix (Corning, NY, USA) and room-temperature IntestiCult^TM^ Organoid Growth Medium (Mouse) (STEMCELL Technologies, Vancouver, Canada) supplemented with penicillin-streptomycin (100 units/100 μg per ml) and Wnt-3A (100 ng/ml, PeproTech, NJ, USA). A small volume of the crypt/medium/Matrigel suspension (50 µl) was pipetted into the center of each well in a 24-well plate (Corning, NY, USA) and incubated at 37 °C for 10 minutes to set the Matrigel. Each well then received 750 µl of room-temperature IntestiCult™ Organoid Growth Medium, and the plate was incubated at 37 °C and 5% CO_2_. The culture medium was changed every other day until the lysis of the organoids and RNA extraction.

For the tumor necrosis factor (TNF)-α stimulation, organoids cultured for 9 days were incubated for 24 hours with 30 ng/ml freshly prepared recombinant mouse TNF-α (R&D systems, MN, USA) and were then immediately lysed for RNA isolation.

### Establishment of the AOM/DSS model

The effect of Cygb deficiency on tumor development in CAC was investigated using the well-established AOM/DSS model^11^. Three individual mice cohorts were used for this study. All mice in the individual cohorts were co-housed to minimize any possible cage-related effects and were weighed once or twice a week. All mice were euthanized by cervical dislocation.

**Figure 1:**
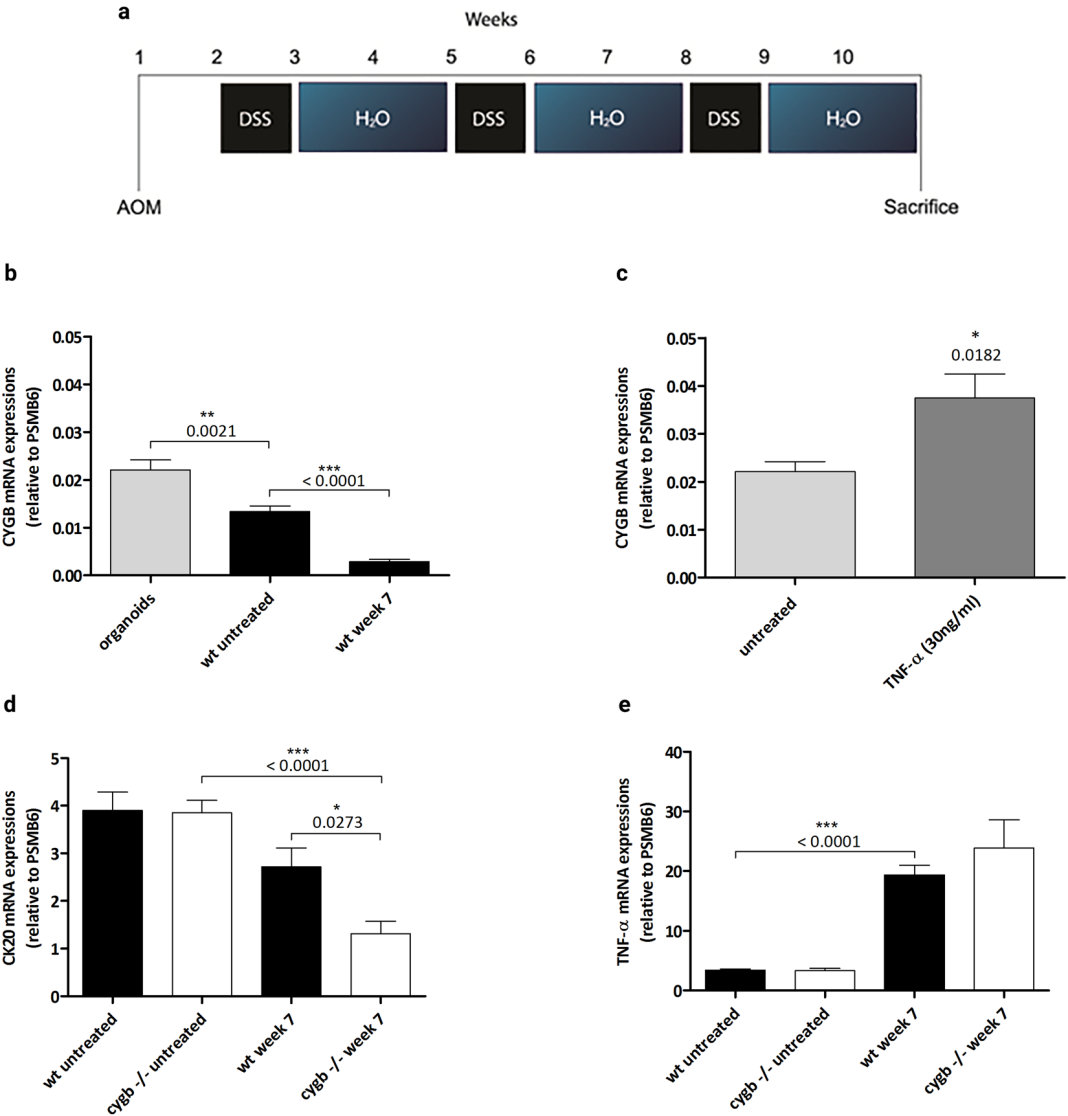
Cygb, Ck20, and TNF-α mRNA expression levels. (**a**) Timeline of the AOM/DSS treatment. Mice were injected i.p. with AOM (7.4 mg/kg), followed by three cycles of 3% DSS in weeks 2, 5 and 8. (**b**) Cygb mRNA expression in cultured organoids from isolated WT colonic epithelial crypts (n = 6), AOM/DSS-treated colonic tissue from week 7 (n = 8) and untreated control WT mice (n = 8). Real-time RT-qPCR was performed to quantify Cygb mRNA expression levels, which were normalized to that of PSMB6. (**c**) Cygb mRNA expression in colonic organoids from WT C57BL/6 mice that were untreated (n = 6) or treated with 30 ng/ml TNF-α (24 h) (n = 6). (**d**) Ck20 mRNA expression in AOM/DSS-treated colonic tissue from week 7, WT (n = 8), Cygb^−/−^ (n = 5) and untreated control WT (n = 8) and Cygb^−/−^ mice (n = 8). (**e**) TNF-α mRNA expression in AOM/DSS-treated colonic tissue from week 7, WT (n = 8), Cygb^−/−^ (n = 5) and untreated control WT (n = 8) and Cygb^−/−^ mice (n = 8). Data are shown as the mean ± SEM. Statistical analysis was performed using Student’s t-test, and P values are shown. P values < 0.05 were considered statistically significant.

1. For the analysis of early colon adenomas, 15 Cygb^−/−^ and 10 WT female mice were injected intraperitoneally (i.p.) with 7.4 mg/kg AOM, followed by one cycle of 3% DSS in week 2 for seven days. All mice were euthanized at the end of in week 5, three weeks after the end of the DSS cycle.
2. For the microarray gene expression analysis of DSS-induced chronic inflammation, 5 Cygb^−/−^ mice and 8 WT female mice were injected i.p. with 7.4 mg/kg AOM, followed by two cycles of 3% DSS for 7 days in weeks 2 and 5. All mice were euthanized in week 7, one week after the end of the second DSS cycle.
3. For the established tumor cohort, 10 Cygb^−/−^ and 12 WT female mice were injected i.p. with 7.4 mg/kg AOM, followed by three cycles of 3% DSS for 7 days/cycle in weeks 2, 5 and 8 (Fig. 1a). All mice were euthanized in week 10, two weeks after the end of the third DSS cycle. In all, 8 Cygb^−/−^ and 8 WT female mice were left untreated to serve as controls.

The experimental protocols were approved by the Danish Veterinary and Food Administration, Ministry of Environment and Food of Denmark and performed according to Danish guidelines.

### RNA extraction, real-time RT-qPCR, hybridization, detection, and signal quantification using whole-genome expression profiling

Colonic tissue harvested from Cygb^−/−^ and WT mice from cohort 2 were stored in RNAlater prior to RNA extraction. Total RNA was extracted using a mirVana miRNA isolation kit (Ambion/Life Technologies, MA, USA) following the manufacturer’s protocol. RNA quality was assessed with an Agilent Bioanalyzer (Palo Alto, CA, USA). For real-time RT-qPCR, first strand cDNA was synthesized with 1 µg of total RNA (or 100 ng of total RNA from colonic organoids) in a total volume of 20 µl using a RevertAid™ H Minus First Strand cDNA Synthesis Kit (Fermentas GmbH, Leon-Rot, Germany) in accordance with the manufacturer’s protocol, with minor modifications. Poly (dT) oligomers (Fermentas) were used as primers. The following PCR primers for Cygb were designed manually: 5′ CCGGGCGACATGGAGATAGA 3′ and 5′ GTCCTCGCAGTTGGCATACAG3′. The primer 3 online facility^12^ was used to select the primers for neurexophilin and PC-esterase domain family member 2 (Nxpe2): 5′ACTGCTCTCTTCGCTTCCAAA 3′ and 5′ TGCGGTTGTTTAAGTGAACCA3′; and PC-esterase domain family member 4 (Nxpe4): 5′CTACGGACCATGTTCAAGTTGC 3′ and 5′ GGACCTCCCTGATTCTCAGTT 3′. The remaining primer pairs were selected using the PrimerBank resource: Psmb6: ID26347247a1; Spry4: ID31543767a1; Ck20: ID21592285a1 and TNF-α: ID7305585a1. Real-time RT-qPCR was performed using a LightCycler 480 system, as previously described^13^. A panel of housekeeping genes, including GAPDH, ACTB, POLII, RPLP0 and PSMB6 was analysed (data not shown). PSMB6 mRNA expression was found to be unaffected by the intestinal inflammation and the most stably expressed throughout the AOM/DSS treatment. This was also confirmed by the microarray gene expression data, where a t-test did not reveal a significant difference in PSMB6 expression between healthy and inflamed tissue. The *in vitro* transcription of labeled probes and hybridization to the Affymetrix mouse gene 2.0 ST array was performed at the Microarray Center (Center for Genomic Medicine, Rigshospitalet, Denmark) according to standard procedures.

Cultured colonic crypt organoids were lysed directly in the wells by adding the lysis buffer provided in the mirVana miRNA isolation kit. The rest of the RNA extraction procedure was performed according to the manufacturer’s protocol.

### Histopathology and immunohistochemistry

Formalin-fixed, paraffin-embedded biopsies from untreated and AOM/DSS-treated (week 7) mice were used. Four micron sections were cut, deparaffinized, and rehydrated. The sections were stained with hematoxylin and eosin (H&E). The histopathological examination of the sections was based on scoring the following 3 parameters^14^. The severity of inflammation was scored from 0–3, as follows: 0, few inflammatory cells in the lamina propria; 1, increased numbers of granulocytes in the lamina propria; 2, confluence of inflammatory cells extending into the submucosa; and 3, transmural extension of the inflammatory infiltrate. Crypt damage was scored from 0–5, as follows: 0, intact crypts; 1, loss of the basal one-third; 2, loss of the basal two-thirds; 3, entire crypt loss; 4, change of epithelial surface with erosion; 5, confluent erosion. Ulceration was scored from 0–3: 0, absence of ulceration; 1, 1 or 2 foci of ulcerations; 2, 3 or 4 foci of ulcerations; 3, confluent or extensive ulceration. These values were added for a maximal score of 11.

A polyclonal rabbit anti-human cytoglobin antibody was raised against bacterially produced human cytoglobin (made in house; code no: 5092/6). As shown in Supplementary Figure S2, the Cygb antibody stains sinusoidal lining cells in the liver of WT mice but not in Cygb^−/−^ mice. This staining pattern is reminiscent of the pattern previously described for a Cygb-specific antibody^15^. For Cygb staining, untreated colons were fixed for 24 hours in phosphate-buffered 10% formalin and then transferred to PBS containing 0.1% sodium azide. For cryosectioning, tissue samples were placed into 30% sucrose in PBS with 0.1% sodium azide for 2 days; then, 10 μm sections were cut using a Leica cryostat and mounted on chrome-gelatin-precoated slides. Immunohistochemistry was performed according to methods previously described by Hundahl *et al*.^16^. In brief, sections (either on slides or free floating) were incubated in 1% perhydrol to block endogenous peroxidase, washed in PBS, preincubated in 1% human serum albumin in PBS, and then incubated with the primary antibody (rabbit anti-human cytoglobin 1:30.000 for mouse tissue or 1:10.000 for human biopsies). The samples were then incubated with the secondary antibody, a biotinylated donkey anti-rabbit (Fab)_2_ antibody at a 1:2000 dilution (Jackson ImmunoResearch Laboratories, PA, USA). Subsequently, the samples were incubated with a horseradish peroxidase (HRP)-conjugated avidin-biotin complex at a 1:200 dilution (Vector Laboratories, CA, USA). As chromogene, either Vector SG substrate kit (Vector Laboratories, CA, USA) or Diaminobenzidine (DAB) was used.

### Macroscopic analysis of early colon adenomas

After euthanization, colons were flushed with PBS through the rectum to smooth the mucosal folds and were then fixed for 3–5 minutes *in situ* via the intraluminal injection of ice-cold 4% paraformaldehyde. The colons were then removed, cut open longitudinally, and pinned to a polyethylene plate with the lumen facing upwards. These samples were stored for an additional 24 hours in 4% paraformaldehyde and thereafter in 70% ethanol until analysis. The colons were washed in distilled water and stained with 0.2% methylene blue in PBS for 30 minutes according to the protocol described by Bird *et al*.^17^ and examined under a stereomicroscope. The total number of adenomas and the number of adenomas larger than 1 mm in diameter were counted. To visualize the mucin-depleted adenomas, the colons were stained using the High Iron Diamine-Alcian Blue (HID-AB) procedure^18^, which stains both sulfomucins and sialomucins. The colons were rinsed for 5 minutes in distilled water and then transferred to 50 ml of a distilled water solution containing 120 mg of N-N′-dimethyl-m-phenylene and 20 mg of N-N′-dimethyl-p-phenylene diamine, to which 1.4 ml of 60% ferric chloride was added. The HID solutions containing the colons were stored in the dark for 18 hours at room temperature. The colons were then rinsed 3 times with distilled water, stained for 30 minutes in 1% alcian blue in 3% acetic acid (pH 2.5) and examined under a stereomicroscope. The total number of adenomas and the number of adenomas larger than 1 mm in diameter were counted again. The distal parts of the colons were severely inflamed, and the mucosa was absent in large areas; therefore, only the proximal half of the colons was included in this analysis. All reagents were purchased from Sigma-Aldrich, Denmark.

### Statistical analysis and sample size estimation

Based on the data from^19^ of the AOM/DSS procedure, the power.t.test function in R was used to calculate the sample size necessary to yield a power of 0.9 for detecting a difference of 5 in tumor counts using the two-sample t-test. The results showed that a sample size of 10 in each group would fulfill these criteria. Mice from one Cygb^−/−^ AOM/DSS cohort and two WT AOM/DSS cohorts were used. No significant differences in the number of tumors were observed between the two Cygb WT cohorts (t-test; P > 0.05).

### Bioinformatics

The data from the Affymetrix mouse gene 2.0 ST array were analyzed using Bioconductor packages^20^ for the R language environment. A single log2 expression measure was calculated for each gene using the RMA algorithm from the Oligo package^21^ and quartile normalization. The log2 gene expression measures and the original CEL files have been deposited in the Gene Expression Omnibus database with the following accession number: GSE86299. A principal component analysis (PCA), an accompanying annotation analysis and a transcription factor binding site overrepresentation analysis were conducted using the pcaGoPromoter package^22^. Initially, a PCA was performed on all intestinal samples representing the following categories: CI_WT: chronic inflammation (week 7) wild type; CI_KO: chronic inflammation Cygb^−/−^ (week 7); UT_WT: untreated wild type; UT_KO: untreated Cygb^−/−^, and the PCA score plot of the first two PCs is depicted in Supplementary Figure S3. Based on a PCA model containing the first three PCs of this initial PCA, a Hotelling T^2^ test was performed comparing CI_WT with CI_KO and UT_WT with UT_KO, respectively. The Hotelling T^2^ test was performed using a multiple linear regression model in R, as previously described^23^. A second PCA was subsequently performed including only the inflamed samples. The score plot of the first two PCs of this PCA is depicted. The most representative gene ontology (GO) terms produced by the GO analysis using the pcaGoPromoter package are depicted in the PCA score plot. These most representative GO terms were derived from plots of the GO trees calculated with the GOtree function in the pcaGoPromoter package. In all, 25 boxes were plotted for the positive direction of the second PC axis (Supplementary Figure S4), and 75 boxes were plotted for the negative direction of the second PC axis (Supplementary Figure S5). The GO terms were selected based on the following criteria: the term selected was either at the third level in a branch that ended at the third level of the GO tree, or the term selected was at the end of a branch with more than three levels. GO terms found only at the second level in the GO tree were not reported. The P values for the GO term overrepresentation analysis were calculated using false discovery rate (FDR) correction for multiple tests^24^. The genes annotated with the GO terms depicted in the PCA score plot were retrieved with the GOhits function in the pcaGopromoter package, and the lists of genes are shown in Supplementary Tables S2 and S3. The promoter transcription factor binding site overrepresentation analysis was performed using the primo function in the pcaGoPromoter package, and the five most significant binding sites are reported in the figure. The complete list of significant binding sites is shown in Supplementary Table S4. The mean differences in expression between WT and Cygb^−/−^ mice were calculated for all genes, and genes with a ≥1.5-fold difference in mean expression were retrieved and subjected to Student’s t-test. The P values were corrected for multiple tests using FDR^24^. The complete lists of up- or down-regulated genes (WT versus Cygb^−/−^ mice) are shown in Supplementary Table S1. The top genes from the lists derived from AOM + DSS treated mice were used for cluster analysis and heatmap visualization. The online resource “HEATMAPPER”^25^ was used employing average linkage and Euclidian distance measuring.

## Results

### Mucosal Cygb mRNA and protein expression in colon organoids and in healthy and inflamed mouse colons

The subcellular expression of Cygb mRNA in the mouse colon was investigated by culturing isolated colonic crypts as organoids. The overall Cygb mRNA expression was low, i.e., the number of transcripts detected in 1000 ng of reverse-transcribed total colonic RNA ranged from 200 to 1500 copies, which is typical for a low-copy number transcript. Following normalization to the Psmb6 gene, it was found that the Cygb mRNA expression was two-fold higher in colonic organoids compared with intact colons (Fig. 1b). Interestingly, the Cygb mRNA expression in cultured organoids increased by almost two-fold after TNF-α stimulation (Fig. 1c). During AOM/DSS-induced inflammation, the Cygb mRNA expression decreased significantly in week 7 (Fig. 1b). These results showed that the highest amounts of Cygb mRNA were found in the intestinal epithelial cells and that the decline in Cygb mRNA expression of the inflamed colons could potentially be due to colonic epithelial cells being lost during severe inflammatory ulceration. To investigate this further, we measured the mRNA expression of the established intestinal epithelial cell marker Cytokeratin 20 (Ck20)^26^ (Fig. 1d). Indeed, we found that CK20 mRNA expression was unaffected in untreated Cygb^−/−^ and WT mice; however, a significant decrease was evident in the inflamed colons of Cygb^−/−^ mice compared with untreated Cygb^−/−^ mice. Interestingly, we also found a significant difference in Ck20 mRNA expression between treated Cygb^−/−^ and WT mice. This result may suggest that Cygb^−/−^ mice are susceptible to more severe inflammation, leading to a significant loss of colonic crypts.

**Figure 2:**
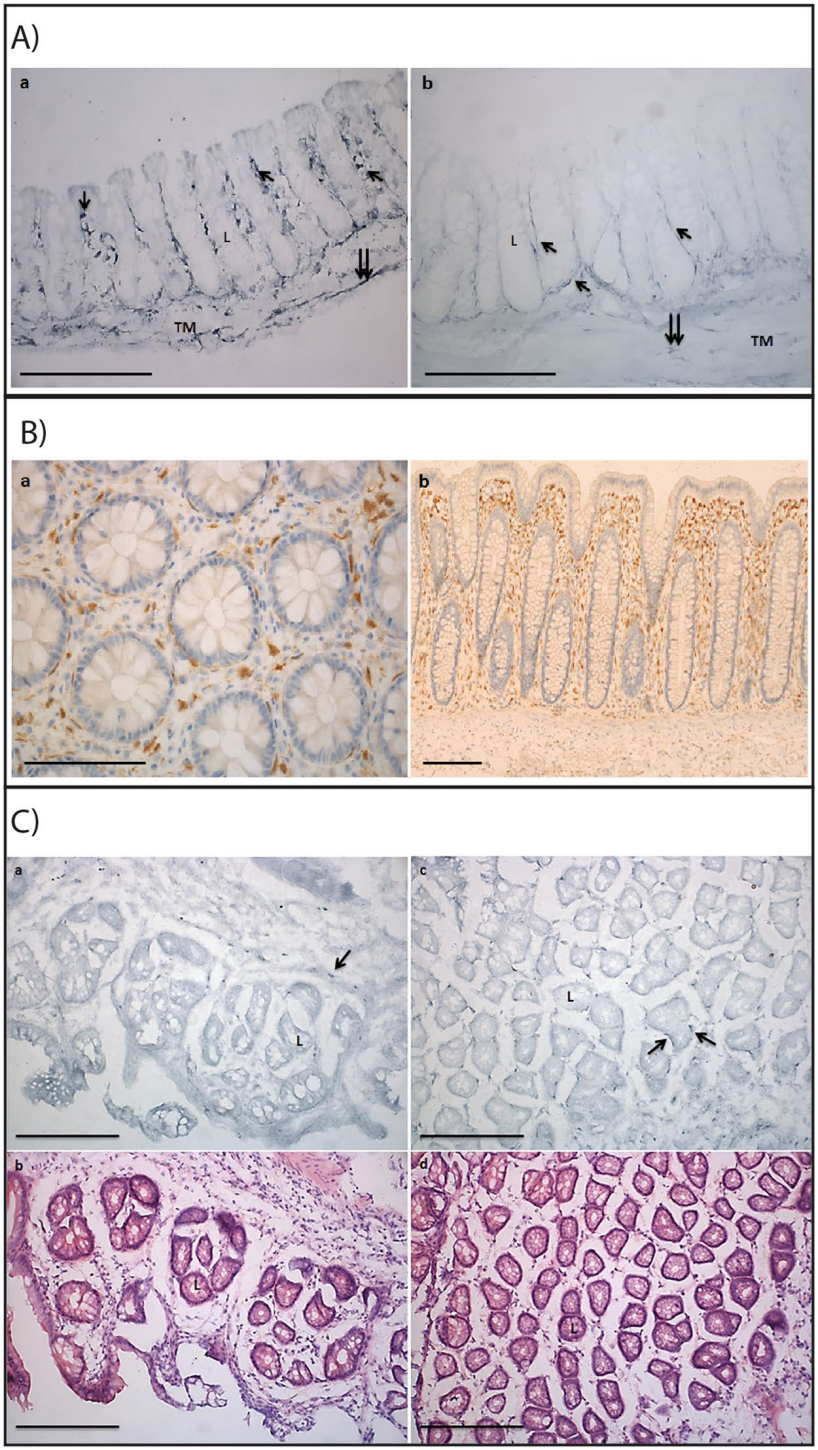
Cytoglobin protein expression in mouse and human colon. (**A**) Cygb-ir in healthy and inflamed C57BL/6 WT mouse colon tissue. (a) Longitudinal section of colonic crypts from untreated mice. (b) Longitudinal section of colonic crypts from week 7 AOM/DSS-treated mice. Single arrows indicate cells with Cygb-ir along Lieberkühn crypts. Double arrows indicate cells with Cygb-ir in tunica muscularis. L: Lieberkühn crypt, TM: tunica muscularis. 20x magnification, scale bars represent 200 µm. (**B**) Cygb expression in normal human colon tissue with hematoxylin counterstaining. No Cygb-ir was observed in the crypts. (a) Cross section, 40x magnification b) Longitudinal section, 5x magnification. Scale bar represents 200 µm. (**C**) Cygb-ir in AOM/DSS-treated C57BL/6 WT mouse colon tissue. (a) Cygb-ir in inflamed area with adenoma. (b) H&E staining of area with adenoma. (c) Cygb-ir in inflamed area without adenoma. (d) H&E staining of area without adenoma. Arrows indicate cells with Cygb-ir. L: Lieberkühn crypt. 20x magnification, Scale bars represent 200 µm.

In the AOM/DSS model, the inflammation observed is lower in the C57BL/6j strain as compared to other mice strains^27^ and samples representative for chronic inflammation were therefore collected at week 7 (after the second DSS cycle) rather than at an earlier time-point. In both healthy and chronic inflamed (AOM/DSS week 7) WT mice (Fig. 2A a-b), colon Cygb-ir is localized to fibroblast-like interstitial cells surrounding the crypts of the mucosal glands, interstitial fibroblast cells of the submucosa, and interstitial Cajal-like cells of the tunica muscularis. However, Cygb-ir seemed to be lower in the inflamed colons, which is in agreement with the Cygb mRNA expressions (Fig. 1b). Moreover, Cygb-ir was not detectable in any cells of the lamina epithelialis. The discrepancy between the mRNA expression and the Cygb-ir results suggests that the Cygb protein is more concentrated in smaller fibroblast-like cells than in larger epithelial cells. Thus, in large epithelial cells, the low Cygb protein expression was below the detection limit. In human intestinal mucosa, Cygb-ir was also found only in the fibroblast-like cells surrounding the crypts (Fig. 2B). In the adenoma and inflamed tissue samples from the AOM/DSS-treated mice, Cygb-ir was again found in fibroblast-like cells scattered around the crypts, albeit much lower than in the healthy colons (Fig. 2C a,c). Tumor cells and epithelial cells showed faint blue staining that could not be clearly distinguished from the background staining resulting from staining with a non-immune serum (not shown).

### Characteristics of colitis development in Cygb-deficient mice

**Figure 3:**
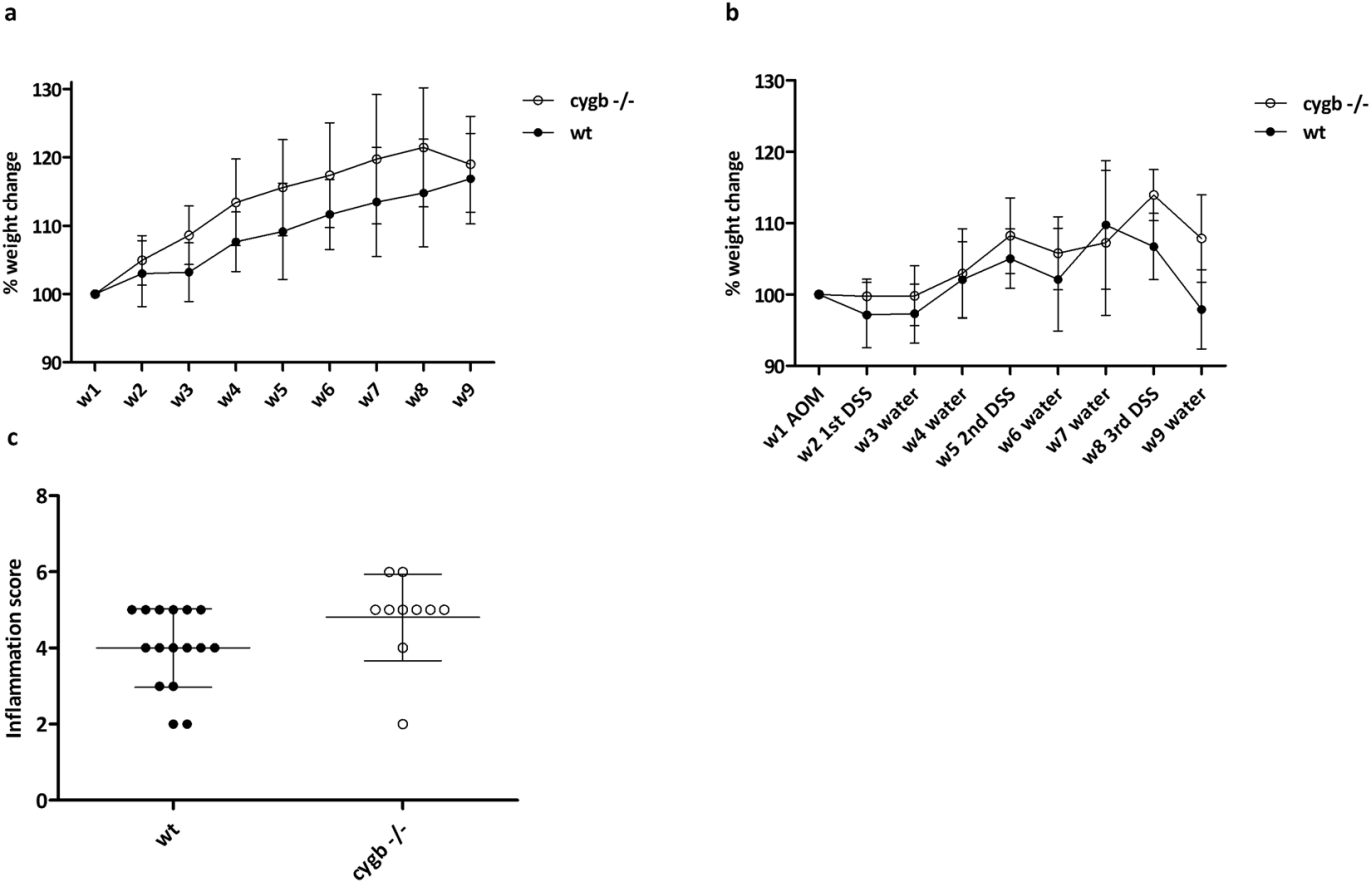
The effect of Cygb on body weight and colon inflammation in AOM/DSS-induced colitis. (**a**) Percentage body weight change relative to week 1 in untreated WT (n = 8) and Cygb^−/−^ mice (n = 8). (**b**) Percentage body weight change relative to week 1 baseline weight. Female WT (n = 10) and Cygb^−/−^ mice (n = 11) after 3 cycles of 3% DSS for 7 days followed by 14 days with tap water. (**c**) Colitis score in non-cancerous tissue in the distal colons after 2 cycles of 3% DSS (week 7) in AOM/DSS-treated WT (n = 8) and Cygb^−/−^ (n = 5) mice. The results are presented as the mean ± SD.

Regarding the untreated littermate controls, we did not observe any differences in body weight change between the WT and Cygb^−/−^ mice during the course of the experiment (Fig. 3a). In addition, we did not observe any significant differences in body weight loss or gain between the two groups upon the administration of AOM and 3 cycles of 3% DSS (Fig. 3b). The same was also the case in other cohorts that were treated with AOM and 1xDSS and 2xDSS before sacrifice (data not shown).

The histological inflammation scores of colon samples showed no significant differences in chronic inflamed Cygb^−/−^ mice treated with AOM and two cycles of 3% DSS (Fig. 3c). The same was also true in mice treated with AOM and only one cycle of 3% DSS (data not shown).

**Figure 4:**
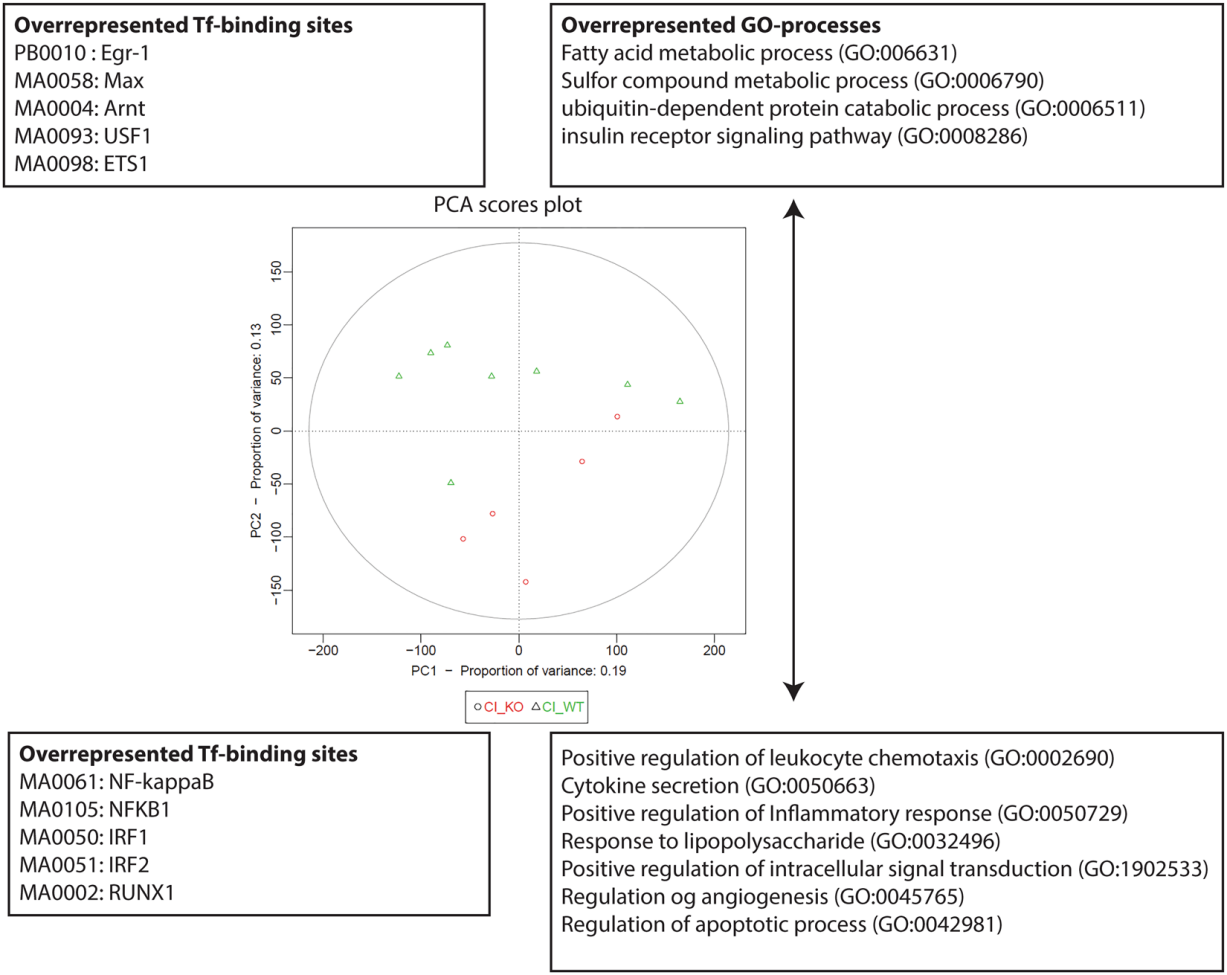
PCA score plot of inflamed colon tissue samples. RNA was extracted from chronically inflamed colons (7 weeks, second DSS cycle) of Cygb^−/−^ mice (chronic inflammation knockout: CI_KO, red circles, n = 5) and from WT mice (chronic inflammation wild type: CI_WT, green triangles, n = 8) and subjected to a microarray analysis (Affymetrix Mouse Gene 2.0 ST array). An unsupervised multivariate analysis followed by GO and promoter overrepresentation analyses were conducted using the pcaGoPromoter package. The score plot of the first two PCs are shown. The variation represented by the second PC (the Y-axis) is correlated with the Cygb genotype. The most important GO terms associated with each direction of the second PC axis are shown together with the most important overrepresented predicted transcription factor binding sites in the promoters of the genes defining either direction of the second PC axis. The results suggest that more-severe inflammation was present in Cygb^−/−^ mice than in WT mice.

The inflammation was also characterized at the molecular level using a bioinformatics strategy based on PCA, as previously demonstrated in human studies of IBD^23^. A Hotelling T^2^ test showed a significant difference (P < 0.05) in the multivariate gene expression pattern between the Cygb^−/−^ and WT mice during inflammation but not in the non-inflamed, untreated state. A PCA score plot shows this difference in the multivariate gene expression pattern in the axis for the second PC of the inflamed samples (Fig. 4). Thus, the WT samples (green triangles) are scattered toward positive values of the second PC axis, whereas the Cygb^−/−^ samples (red circles) are scattered toward negative values of this axis. The GO analysis of genes driving the samples toward positive or negative values, respectively, along the second PC axis showed that genes annotated with the following terms defined the negative direction along the Y-axis in the PCA score plot: leukocyte chemotaxis; cytokine secretion; positive regulation of the inflammatory response; response to lipopolysaccharide; positive regulation of intracellular signal transduction; regulation of angiogenesis; and regulation of the apoptotic process (Fig. 4). Thus, samples with high expression levels of such genes are plotted with negative values on the Y-axis. Such samples were mostly the Cygb^−/−^ samples with chronic inflammation. In contrast, the genes that defined the positive direction of the second PC axis (the Y-axis) were annotated with the following GO terms: fatty acid metabolic process; sulfur compound metabolic process; ubiquitin-dependent protein catabolic process; and insulin receptor signaling pathway. Thus, samples with high expression levels of such genes are plotted with positive values on the Y-axis. Such samples were mostly WT samples with chronic inflammation.

The conclusion of the GO analysis was that in the analyzed samples with chronic inflammation, the Cygb^−/−^ samples were more inflamed, as determined by the annotation analysis of the genes expressed at increased levels in these samples compared with the WT samples. To supplement the GO analysis, an analysis of predicted transcription factor binding site overrepresentation in the promoters for the genes defining the Y-axis in either direction was conducted. The results showed that the promoters of the genes defining the negative direction of the Y-axis had an overrepresentation of predicted transcription factor binding sites for nuclear factor-κB, interferon response factors 1 and 2, and runt-related transcription factor 1. These findings further support the conclusion that the Cygb^−/−^ samples were more inflamed based on their expression of inflammation-related genes.

### Overall characterization of genes with increased expression in the inflamed colons of Cygb^−/−^ mice compared with WT mice

**Figure 5:**
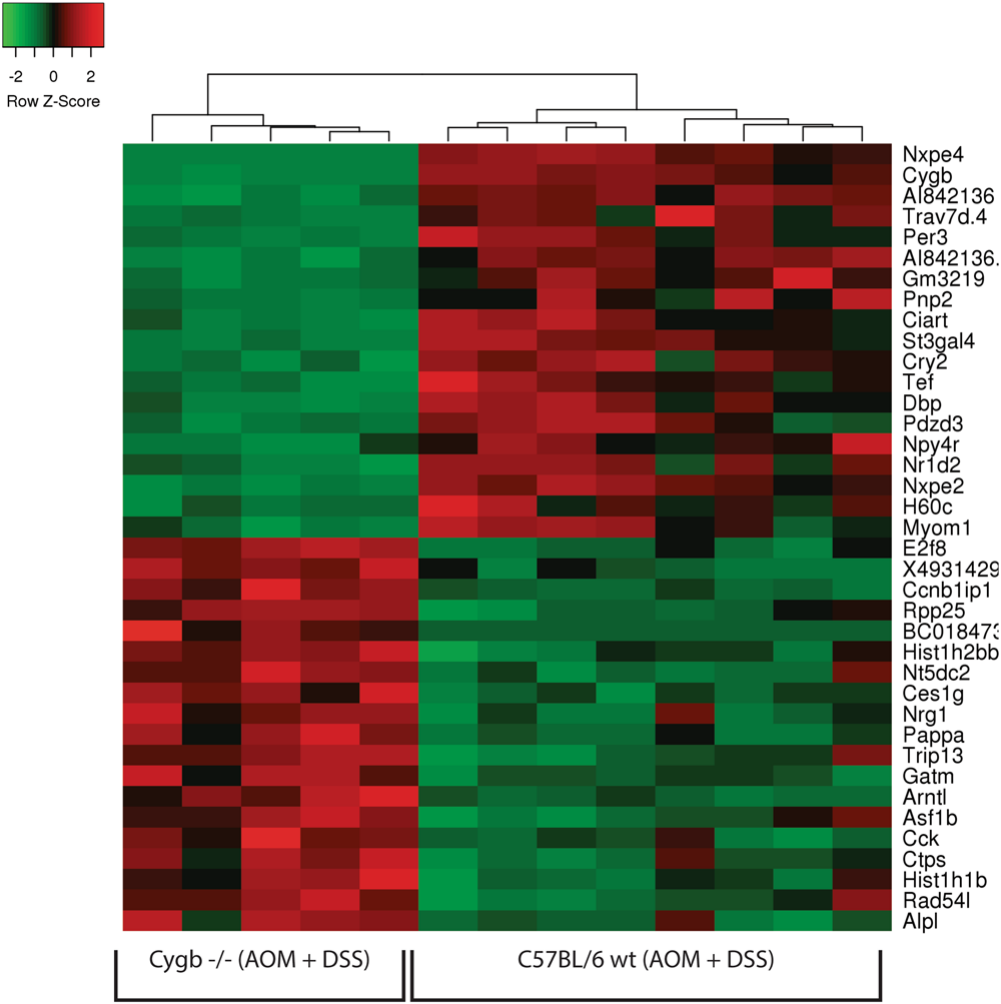
A heatmap depicting the relative expression of the top list of genes (rows) that are either highest expressed (red color) in the wild type mice or in the Cygb^−/−^ mice during AOM/DSS-induced colitis. Gene symbols are shown to the right. Each column represents an individual mouse.

A direct comparison of genes that displayed at least a 1.5-fold mean difference in expression between the inflamed colonic samples from WT and Cygb^−/−^ mice was conducted using the t-test (Supplementary Table S1). The top genes were used for cluster analysis and heatmap visualization (Fig. 5). Eighty genes displayed more than 1.5-fold higher mean expression levels (FDR < 0.05) in the inflamed colon samples from Cygb^−/−^ mice compared with the inflamed colon samples from WT mice. Eighty-two genes displayed more than 1.5-fold higher mean expression levels (FDR < 0.05) in the WT samples compared with the Cygb^−/−^ samples. Notably, the Cygb gene itself exhibited significantly higher expression in the WT samples, indicating that the Cygb mRNA lacking the deleted exon 2 in the Cygb^−/−^ mice might be unstable.

In accordance with the GO analysis, many genes that are markers of inflammation displayed increased expression in the inflamed colons of Cygb^−/−^ mice compared with those of WT mice. The most noteworthy were the genes for chemokine (C-C motif) ligand 9 (CCL9), chemokine (C-X-C motif) ligand 1 (CXCL1), CXCL2, and CXCL10, matrix metalloproteinase (MMP) 3, MMP8, MMP9, MMP10, and MMP13, and interleukin (IL)-17a. In addition, it should be noted that a non-coding transcript (cDNA sequence BC018473) was up-regulated by 23-fold in the inflamed samples from Cygb^−/−^ mice. However, this non-coding transcript does not have a human ortholog and was therefore not further investigated in the present work. In the following sections, a more detailed analysis of the subsets of relevant genes is presented.

### Cygb is required for the expression of IBD- and UC-associated genes during DSS-induced colitis

**Figure 6:**
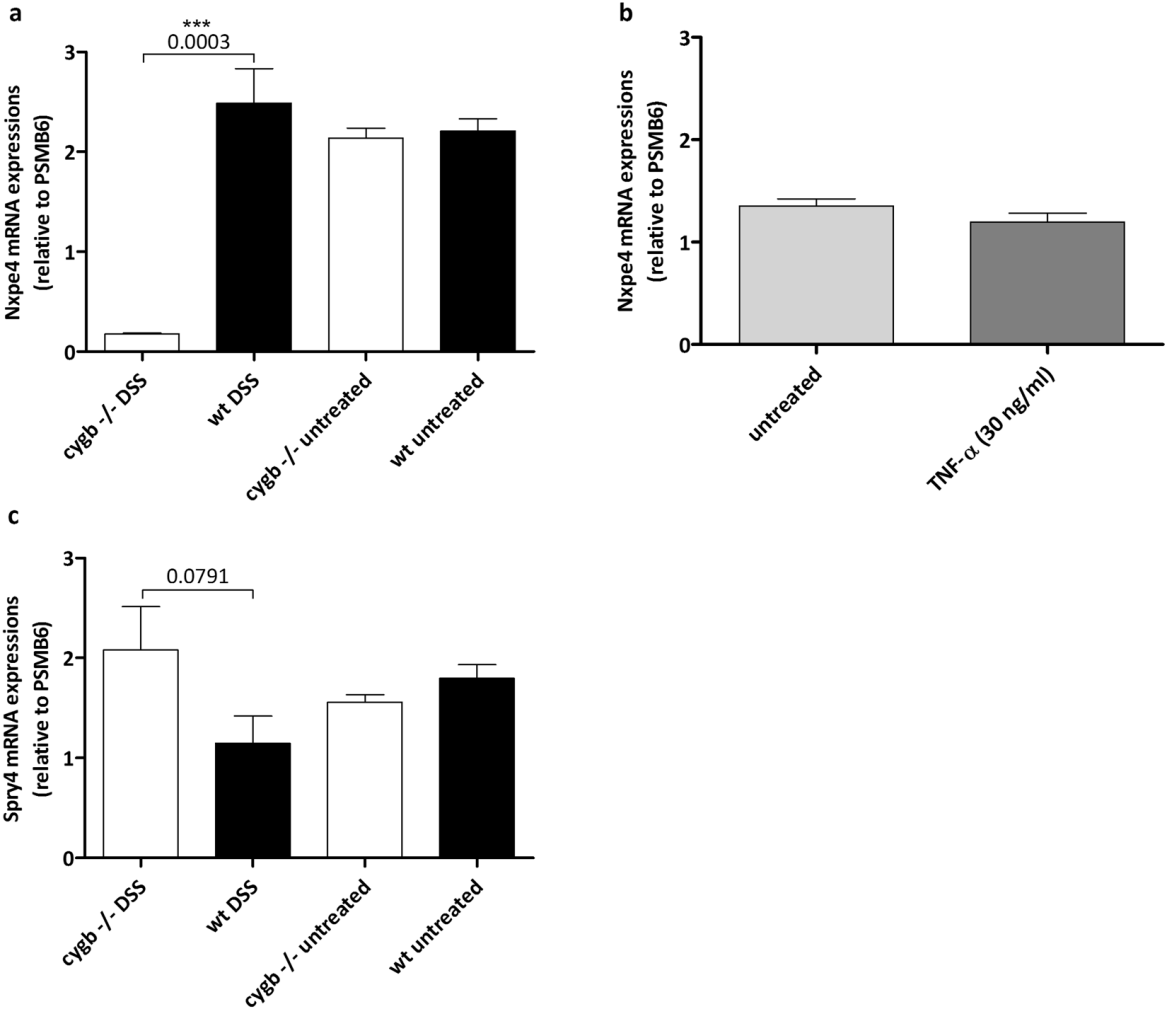
Nxpe4 and Spry4 mRNA expression levels. (**a**) Nxpe4 mRNA expression in colon tissue from week 7 AOM/DSS-treated C57BL/6 mice, WT (n = 8), Cygb^−/−^ (n = 5), untreated control WT (n = 8) and Cygb^−/−^ mice (n = 8). (**b**) Nxpe4 mRNA expression in cultured organoids from isolated WT colonic epithelial crypts, untreated colonic epithelial crypts (n = 6) and colonic epithelial crypts treated with 30 ng/ml TNF-α (24 h) (n = 6). (**c**) Spry4 mRNA expression in colon tissue from week 7 AOM/DSS-treated C57BL/6 mice, WT (n = 8), Cygb^−/−^ (n = 5), untreated control WT (n = 8) and Cygb^−/−^ mice (n = 8). Data are shown as the mean ± SEM. Statistical analysis was performed using Student’s t-test, and P values are shown. P values < 0.05 were considered statistically significant.

The lists of genes differentially expressed between the inflamed colons from Cygb^−/−^ mice and WT mice were searched for genes associated with IBD as determined in a recent genome-wide association study^28^. This search led to the identification of the Nxpe2 and Nxpe4 genes and the Sprouty 4 gene (Spry4). Based on the microarray data presented in the present study, Nxpe2 and Nxpe4 are expressed at higher levels (more than 5- and 16-fold, respectively) in the inflamed colons of WT mice than in those of Cygb^−/−^ mice. Interestingly, the expression levels of these genes are the same in non-inflamed colons from WT and Cygb^−/−^ mice. The expression of these genes appears to depend on the presence of Cygb only during inflammation. Additionally, Spry4 has been linked to IBD^28^, and in our microarray analysis, Spry4 was up-regulated in the inflamed colons from Cygb^−/−^ mice compared with those from WT mice (1.6-fold, FDR < 0.05). These findings were verified by RT-qPCR. By this analysis, Nxpe4 was confirmed to be expressed at much higher levels in chronically inflamed colons from WT mice than in those from Cygb^−/−^ mice (Fig. 6a). Furthermore, Nxpe4 expression was also evident in the cultured organoids of the colonic crypts isolated from WT mice (Fig. 6b). This expression was not affected by TNF-α, which was in agreement with the Nxpe4 mRNA expression levels observed in the DSS-treated and untreated WT colons. However, the RT-qPCR primers failed to amplify Nxpe2. The RT-qPCR results showed that the Spry4 mRNA expression trend was comparable to that revealed by the microarray analysis (1.8-fold higher in inflamed Cygb^−/−^ colons), but the difference was not significant (Fig. 6c). In addition, Spry4 mRNA expression did not change after TNF-α stimulation in the cultured organoids of the colonic crypts isolated from WT mice (data not shown).

### Modest changes in the expression of genes involved in oxidative stress responses

GO terms related to oxidative stress were found among the GO terms that were overrepresented in the annotation of genes defining the negative direction of the second PC axis. However, the selection criteria for the representative GO terms shown in Fig. 4 did not identify these genes annotated with GO terms related to oxidative stress. This is partly because the P values reported for these terms were higher and partly because fewer terms related to oxidative stress than to inflammation were revealed by the analysis. The following GO terms were significantly associated with Cygb-dependent expression during DSS-induced colitis: GO:0000302: response to reactive oxygen species (ROS); GO:0072593: ROS metabolic process; GO:0006809: NO biosynthetic process; and GO:0045429: positive regulation of NO biosynthetic process. Table 1 shows a selected list of genes from the analysis; the cyclooxygenase (COX)-2 gene (Ptgs2: prostaglandin-endoperoxide synthase 2) is the only gene that was up-regulated more than 2-fold in the inflamed colons from Cygb^−/−^ mice. All other genes displayed less than 1.7-fold differences in expression.

**Table 1:** Genes with differential expression during inflammation and encoding proteins involved in the metabolism of reactive oxygen species.

Symbol	Gene Name	^1^Mean log_2_ wt (n = 8)	^1^Mean log_2_ Cygb^−/−^(n = 5)	Fold up	p-value
Cycs	cytochrome c, somatic	10.8 ± 0.1	11.0 ± 0.04	1.2	0.01
Ddah1	dimethylarginine dimethylaminohydrolase 1	8.8 ± 0.4	9.3 ± 0.2	1.3	0.03
Gpx3	glutathione peroxidase 3	6.2 ± 0.2	6.6 ± 0.2	1.3	0.01
Hmox1	heme oxygenase (decycling) 1	7.6 ± 0.3	8.3 ± 0.3	1.6	0.01
Ptgs2	prostaglandin-endoperoxide synthase 2	8.0 ± 0.7	9.3 ± 1.0	2.5	0.03
Sod2	superoxide dismutase 2, mitochondrial	9.7 ± 0.1	9.9 ± 0.2	1.2	0.04
Sod3	superoxide dismutase 3, extracellular	7.7 ± 0.2	8.1 ± 0.3	1.3	0.04

^1^Mean log_2_ expression measure ± sd.

### Cygb deficiency and the development of early adenomas

**Figure 7:**
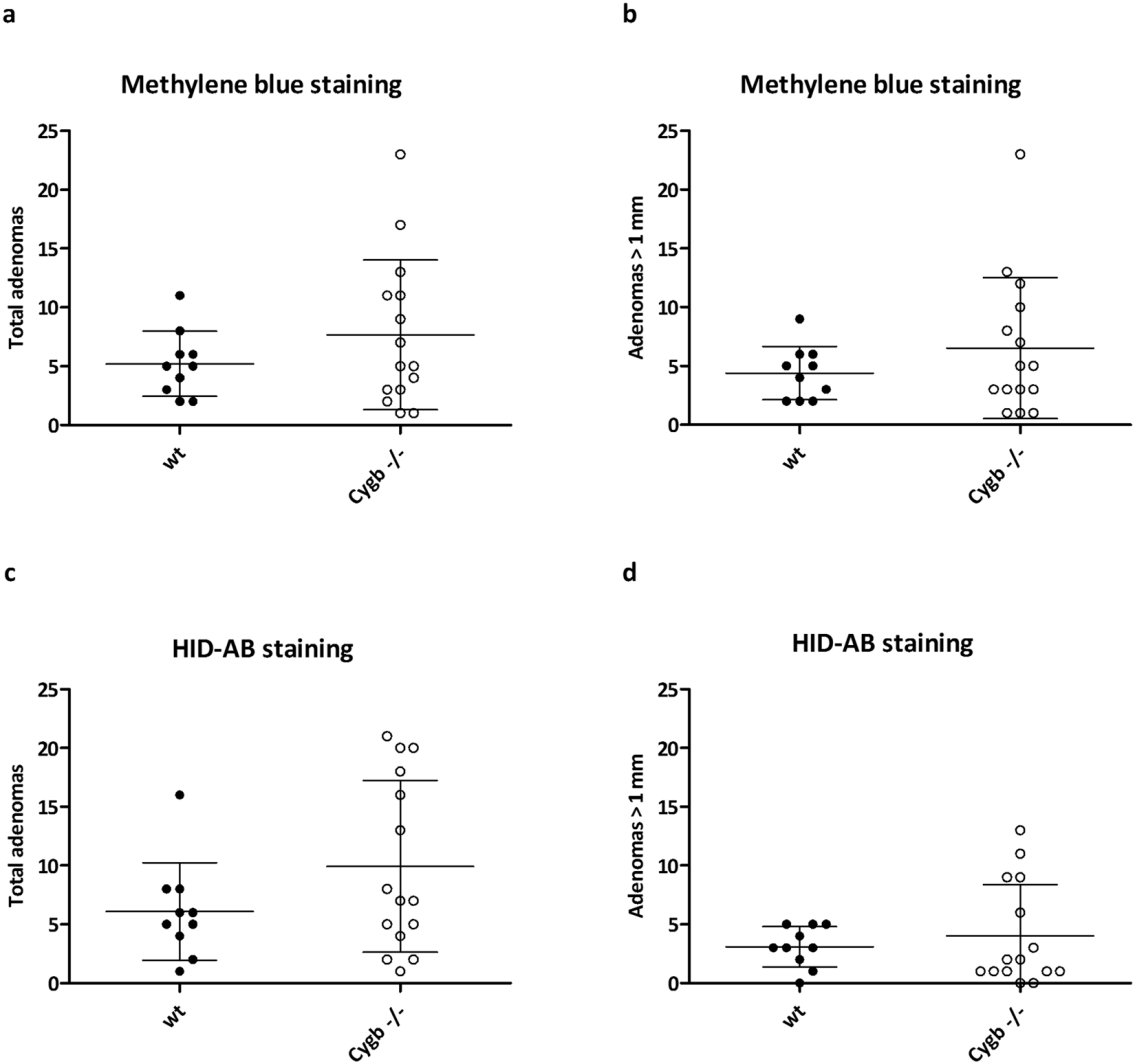
Early adenomas in Cygb-deficient mice. Colons were pinned onto a plate, stained with methylene blue, and then total adenomas (**a**) and adenomas >1 mm (**b**) were counted under a stereomicroscope. After this procedure, colons were stained with HID-AB, and both total adenomas (**c**) and adenomas >1 mm (**d**) were counted again. Each dot represents an individual mouse. The results are presented as the mean ± SD. No significant differences were found between WT and Cygb^−/−^ mice. The experiment was initiated by AOM injection followed by one cycle of 3% DSS in week 2 for seven days. All mice were euthanized at the end of week 5, three weeks after the end of the DSS cycle.

When the proximal mucosal surface of the methylene blue-stained colon was visualized with the stereomicroscope, we detected a mean ± standard error of the mean (SEM) = 8 ± 1.6 adenomas/mid colon in the Cygb^−/−^ group n = 15, and 5 ± 0.9 in the WT group n = 10; however, these values were not significantly different (Fig. 7a). Almost all counted adenomas (85% in both groups) were adenomas >1 mm (Fig. 7b). In the Cygb^−/−^ group, we detected 6 ± 1.5 vs. 4 ± 0.7 in the WT group, and this difference was also not significant. After staining with HID-AB, we found that very small microadenomas were also visible (Fig. 7c,d); therefore, the total number of adenomas increased in both groups; yet, the means remained similar (10 ± 1.9 vs. 6 ± 1.3).

### Cygb deficiency influences development of colitis-associated tumors

**Figure 8:**
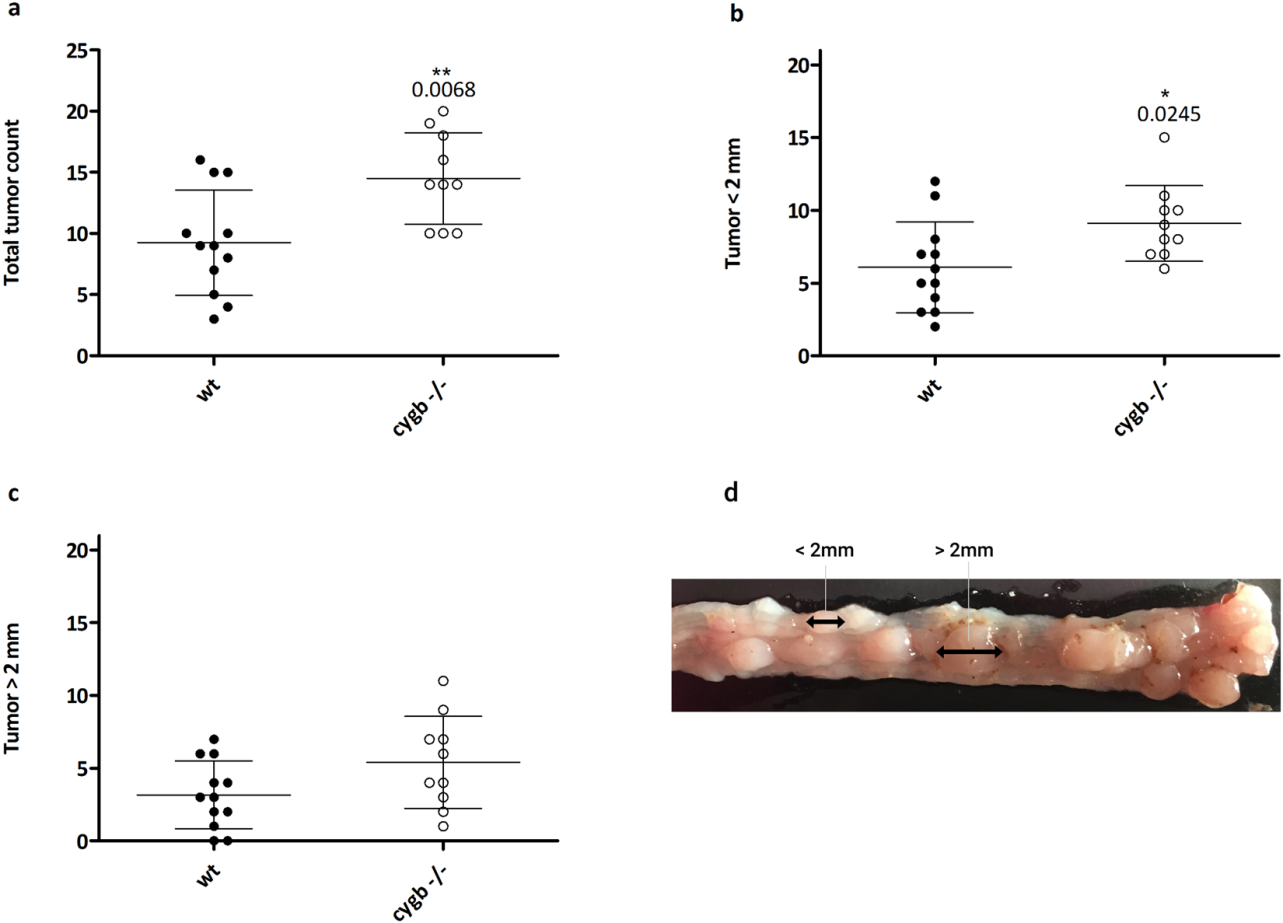
Tumorigenesis in Cygb-deficient mice. (**a**) Comparison of the total numbers of macroscopic tumors in WT (n = 12) and Cygb^−/−^ mice (n = 10). (**b**) Tumors <2 mm in WT and Cygb^−/−^ mice. (**c**) Tumors >2 mm in WT and Cygb^−/−^ mice. (**d**) A representative image of distal colonic tumors (<2 mm and >2 mm) from an AOM/DSS-treated mouse colon. Each dot represents an individual mouse. The results are presented as the mean ± SD. Statistical analysis was performed using Student’s t-test, and P values are shown. The experiment was initiated by AOM injection, followed by three cycles of 3% DSS for 7 days/cycle in weeks 2, 5 and 8. All mice were euthanized in week 10, two weeks after the end of the third DSS cycle.

Cygb-deficient mice developed a significant increased number of colonic tumors (mean ± standard error of the mean (SEM) = 14.5 ± 1.186, n = 10) compared with WT mice (mean ± SEM = 9.25 ± 1.244, n = 12) following the AOM/DSS regimen, primarily in the distal part of the colon (Fig. 8a). Moreover, when tumors were subcategorized according to size (Fig. 8b–d), Cygb^−/−^ mice exhibited significantly more tumors <2 mm than did WT mice (Fig. 8b). While not significant, the Cygb^−/−^ mice tended to develop an increased number of large tumors (>2 mm) compared with WT mice (Fig. 8c). Taken together, these data show that the inactivation of Cygb promotes increased tumorigenesis in AOM/DSS-treated mice, suggesting that Cygb has tumor-suppressive characteristics.

### Selected CAC-associated genes affected by Cygb expression during DSS-induced colitis

The list of genes (Supplementary Table S1) displaying more than 1.5-fold increases in expression in the inflamed colons from Cygb^−/−^ mice compared with the inflamed colons from WT mice was searched for genes that might affect tumor development. Known cancer-associated genes, cell cycle-related genes or genes encoding growth factor receptors were specifically included in the search. Based on this search, the following genes were emphasized: Ptgs2, cyclin B1 interacting protein 1 (Ccnb1ip1), E2F transcription factor 8 (E2f8), epiregulin (Ereg), amphiregulin (Areg) and neuregulin 1 (Nrg1).

## Discussion

Inflammation-associated CRC is a critical concern for patients with UC, and understanding the genes involved is of high importance for future therapeutic developments and for the care of these patients. Here, we investigated the role of Cygb in a murine model of inflammation-associated CRC and observed at the molecular level that Cygb-deficient mice developed more severe inflammation, which seemed to be of importance for the advancement of established dysplasia, as Cygb-deficient mice also developed an increased number of tumors. In contrast, no significant difference in the number of microadenomas was detected between Cygb^−/−^ and WT mice (Fig. 7), suggesting that in the AOM/DSS model, Cygb does not affect tumor initiation. However, it should be noted that AOM is a very potent mutagen, which probably could mask a weak effect of Cygb as a scavenger of genotoxic ROS in the tumor initiation phase.

Interestingly, during DSS-induced colitis, a change in the expression of three genes associated with IBD in a recent genome-wide association study^28^ was observed when comparing WT with Cygb^−/−^ mice; these were the Nxpe2, Nxpe4 and Spry4 genes. In humans, Nxpe2 and Nxpe4 are located on chromosome 11 (11q23.2) and have been identified as UC-associated candidate genes^28^. The functions of Nxpe2 and Nxpe4 remain unknown, but they are secreted proteins with a domain displaying similarity with members of the neurexophilin protein family^29^. Here, we show that they are highly dependent on Cygb during colonic inflammation, but the underlying mechanisms require further elucidation. Sprouty proteins regulate receptor tyrosine kinase signaling, mostly as inhibitors, by suppressing the linked mitogen-activated protein kinase signaling pathways^30^. They are involved in the regulation of tumorigenesis and have been reported to be aberrantly expressed in different types of human cancer^31^. In a human ovarian cancer cell line, Spry4 overexpression enhanced Areg-induced cell invasion^32^. In the same study, treatment with Areg up-regulated Spry4 by activating the ERK1/2 signaling pathway. Interestingly, Areg was also found to be up-regulated in this study. Moreover, Spry4 has indeed been reported to be up-regulated during cellular hypoxia^33^.

Cygb is expressed in specific neurons of the central nervous system^16^ and in peripheral fibroblasts^34^. Cygb is thought to be expressed in most cells, but the most convincing analyses of expression have been reported from distinct hepatic cells and connective and nervous tissue (reviewed in^2^). Nonetheless, few reports on intestinal expression are available, and these reports have demonstrated expression in rectal subepithelial myofibroblasts^35^. Interestingly, a decrease in Cygb-positive subepithelial myofibroblasts, correlating with α smooth muscle actin-positive and heat shock protein 47-positive cells, was significant in long-term UC with neoplasia^35^. The data presented here show clear Cygb protein expression in fibroblast-like cells surrounding the crypts. However, Cygb mRNA expression was also found in the colonic crypt cells, whereas no clear Cygb protein expression could be demonstrated by immunohistochemistry, suggesting that the Cygb protein concentration is low in epithelial cells. Our finding that TNF-α increases Cygb mRNA expression in colonic epithelial cells might support a role for Cygb as a cytoprotective protein in epithelial cells during inflammation.

In addition, several reports have indicated the hypoxia-dependent regulation of Cygb expression in various tissues, including tumor tissues^36^. On the contrary, the hypoxic microenvironment may be beneficial for tumor development^37^. Thus, it can be speculated that Cygb can function as a tumor suppressor also by counteracting hypoxia. Nevertheless, it seems that Cygb expression in cancer depends on different conditions in the microenvironment, including not only the available level of oxygen and the inflammatory status but also the cancer cell type and the tumor progression stage. Mechanistically, in addition to the suggested role of Cygb in ROS detoxification under oxidative stress, a tumor-suppressive role has been linked to the DNA damage response and the maintenance of genome integrity via association with p53^38^. Cygb expression was shown to be induced in response to genotoxic stress and was associated with p53 by stabilizing and prolonging its half-life, resulting in G1 cell cycle arrest. This association of Cygb with p53 and the prevention of its rapid ubiquitination, which abrogated the cell cycle arrest imposed by DNA damage, was further emphasized in Cygb-deficient cells^38^. In human epidermal keratinocytes, Cygb was identified as a novel direct transcriptional target of ΔNp63, a protein predominantly expressed in proliferative epithelial compartments and involved in epidermal oxidative stress during physiological aerobic metabolic processes^39^. In the same study, knocking down Cygb in H_2_O_2_-treated keratinocytes resulted in increased ROS levels and oxidative stress-induced apoptosis.

In addition to the diminished ROS scavenging in the tumor microenvironment and the increased hypoxic environment leading to accelerated tumor progression, our data indicate that the tumor promotion in Cygb-deficient mice may also be attributed to an increased inflammatory environment, further supporting inflammation-associated cancer development. Hence, we observed an overall increase in the level of colitis as measured by a genome-wide expression analysis. Thus, inflammation-related genes, such as those for CCL9, CXCL1, CXCL2, CXCL10, MMP3, MMP8, MMP9, MMP10, MMP13 and IL-17a, demonstrated increased expression in Cygb-deficient mice during AOM/DSS-induced colitis. A common feature of these genes is their perpetuation of mucosal inflammation in IBD. IL-17a is known to be implicated in autoimmunity and the pathogenesis of IBD. In addition to stimulating the production and activation of MMPs, IL-17a induces the secretion of an array of pro-inflammatory cytokines, such as IL-6, TNF-α, IL-1β and several chemokines^40^.

Furthermore, altered chemokine secretion is a key player in initiating and maintaining the inflammatory response in IBD. CXCL1 is highly expressed in human colorectal tumors, and both CXCL1 and CXCL2 are involved in IBD and believed to promote tumor angiogenesis by directly activating their shared receptor, CXCR2, on endothelial cells^41^. The serum levels of the neutrophil chemoattractant CXCL1 are elevated in IBD patients^42^ and reduced during remission after the initiation of therapy^43^. The high correlation of the CXCL1 level with the disease grade suggests that it could be a useful biomarker of IBD activity. CXCL1-deficient mice exhibit increased susceptibility and exaggerated responses to DSS-induced colitis, with a complete loss of gut integrity and predominantly mononuclear infiltrations with very few infiltrating neutrophils^44^. Thus, CXCL1 seems to be a crucial component in both the recruitment of neutrophils to the intestine and the restoration of mucosal barrier integrity during an inflammatory response.

The mucosal up-regulation of chemokines in IBD is correlated with disease activity and may further enhance the tumor-promoting microenvironment. CCL9 seems to be implicated in colon tumor metastasis, as it was increased in the tumor epithelium in a cis-Apc/Smad4 mouse model of spontaneous CRC progression that showed marked invasion^45^. In the same model, the lack of C-C motif chemokine receptor 1, the cognate receptor for CCL9, prevented the accumulation of MMP-expressing cells and significantly suppressed tumor invasion into the smooth muscle layer.

It has been reported that interferon-y-induced CXCL10 regulates crypt cell proliferation during DSS-induced colitis and that the neutralization of CXCL10 protected mice from epithelial ulceration by promoting crypt cell survival^46^. In addition, the abrogation of colitis in IL-10^−/−^ mice with the deletion of CXCL10 further emphasized its role in IBD^47^. Indeed, UC patients have been reported to have higher mucosal expression of CXCL10^48^, and increased CXCL10 serum levels have also been shown in IBD patients^49^. Blocking CXCL10 with human anti-CXCL10 monoclonal antibodies has shown promising results in a clinical trial with UC patients, but further dose-response studies are required^50^.

The proteases most predominantly expressed in the gut mucosa during active IBD are MMPs, and this connection has been well described. The increase has long been associated with the destructive properties of IBD, providing a basis for the colitis-CRC link. Activated MMPs, including MMP3, MMP8, MMP9 and MMP13, have all been reported to be elevated in the inflamed mucosa of both IBD patients and in experimental DSS-induced colitis^51–53^. Furthermore, a previous study utilizing the AOM/DSS model demonstrated a down-regulation of three murine microRNAs, miR-128, miR-134 and miR-330, which regulate MMP3, MMP10 and MMP13, respectively, leading to an up-regulation of these proteases in the inflamed tissues^54^. MMP9, one of the most abundantly expressed proteases in the inflamed bowel of colitis patients^51^, is also associated with increased levels of CXCL1 and seems to be involved in increased epithelial tight junction permeability during DSS-induced colitis^55^. Neutrophils infiltrating the mucosa are believed to be the likely source of this protease^51^. Furthermore, a recent study suggests that MMP3 is responsible for the proteolytic degradation that contributes to the non-responsiveness to anti-TNF agents in IBD patients^56^.

In addition to the above-mentioned genes, we also found that cancer-associated genes, cell cycle-related genes and genes encoding growth factor receptors were up-regulated in Cygb-deficient mice. As mentioned above, Ptgs2, also known as COX-2, showed more than a 2.5-fold increase in expression in the inflamed colons from Cygb^−/−^ mice. Ptgs2 is a known chemoprevention target for CRC, and its expression is increased in types of human gastrointestinal cancer^57^. Ccnb1ip1 showed an almost 3-fold increase in expression in the inflamed colons from Cygb^−/−^ mice. Ccnb1ip1 encodes E3 ubiquitin ligases, and its expression decreases cyclin B protein levels. E2f8 showed a 1.6-fold increase in expression in the inflamed colons from Cygb^−/−^ mice. E2f8 has been reported to show increased expression in lung cancer and to be important for the growth of lung cancer cells. Ereg, Areg and Nrg1 showed 2-, 1.6- and 1.6-fold increases in expression in the inflamed colons from Cygb^−/−^ mice, respectively. Ereg, Areg and Nrg1 are all ligands to members of the ErbB/HER growth factor receptor family, and all three proteins have been implicated in cancer development^58^.

In conclusion, we have shown that Cygb deficiency exacerbated AOM/DSS-induced inflammation and augmented the establishment of colonic tumors. We also identified two functionally unknown genes, Nxpe2 and Nxpe4, which are highly dependent on Cygb expression. The up-regulation of leukocyte chemoattractants, genes involved in tissue remodeling and destruction, and genes known to be involved in cancer development strongly implies that the lack of functional Cygb amplifies the intestinal damage induced by AOM/DSS. This effect could possibly be mediated by a lack of toxic metabolite scavenging, which may increase epithelial cell death and mucosal inflammation. Thus, the enhanced inflammatory response may be a critical factor leading to a significantly higher propensity for malignant development in the distal colon, suggesting a role for Cygb in suppressing established microadenomas.

## Electronic supplementary material

Supplementary figures and tables
