# Supplementary material for: Cytoglobin affects tumorigenesis and the expression of ulcerative colitis-associated genes under chemically induced colitis in mice
Source: Sci Rep. 2018 May 2;8:6905. doi: 10.1038/s41598-018-24728-x (PMC5931983; doi:10.1038/s41598-018-24728-x)
Supplement: Supplementary file 1 — Supplementary figures and tables [file 41598_2018_24728_MOESM1_ESM.pdf]

Cytoglobin affects tumorigenesis and the expression of ulcerative colitis-associated genes under chemically induced colitis in mice

Mohammad Yassin[1], Hannelouise Kissow[2], Ben Vainer[3], Philomeena Daphne Joseph[4], Anders Hay-Schmidt[4*], Jørgen Olsen[1*] and Anders Elm Pedersen[5*]

[1] Department of Cellular and Molecular Medicine, Faculty of Health and Medical Sciences, University of Copenhagen, Copenhagen, Denmark

[2] Department of Biomedical Sciences and NNF Center of Basic Metabolic Research, Faculty of Health and Medical Sciences, University of Copenhagen, Copenhagen, Denmark

[3] Department of Pathology, Rigshospitalet, University of Copenhagen, Copenhagen, Denmark

[4] Department of Neuroscience and Pharmacology, Faculty of Health and Medical Sciences, University of Copenhagen, Copenhagen, Denmark

[5] Department of Immunology and Microbiology, Faculty of Health and Medical Sciences, University of Copenhagen, Copenhagen, Denmark

*These authors contributed equally as senior authors.

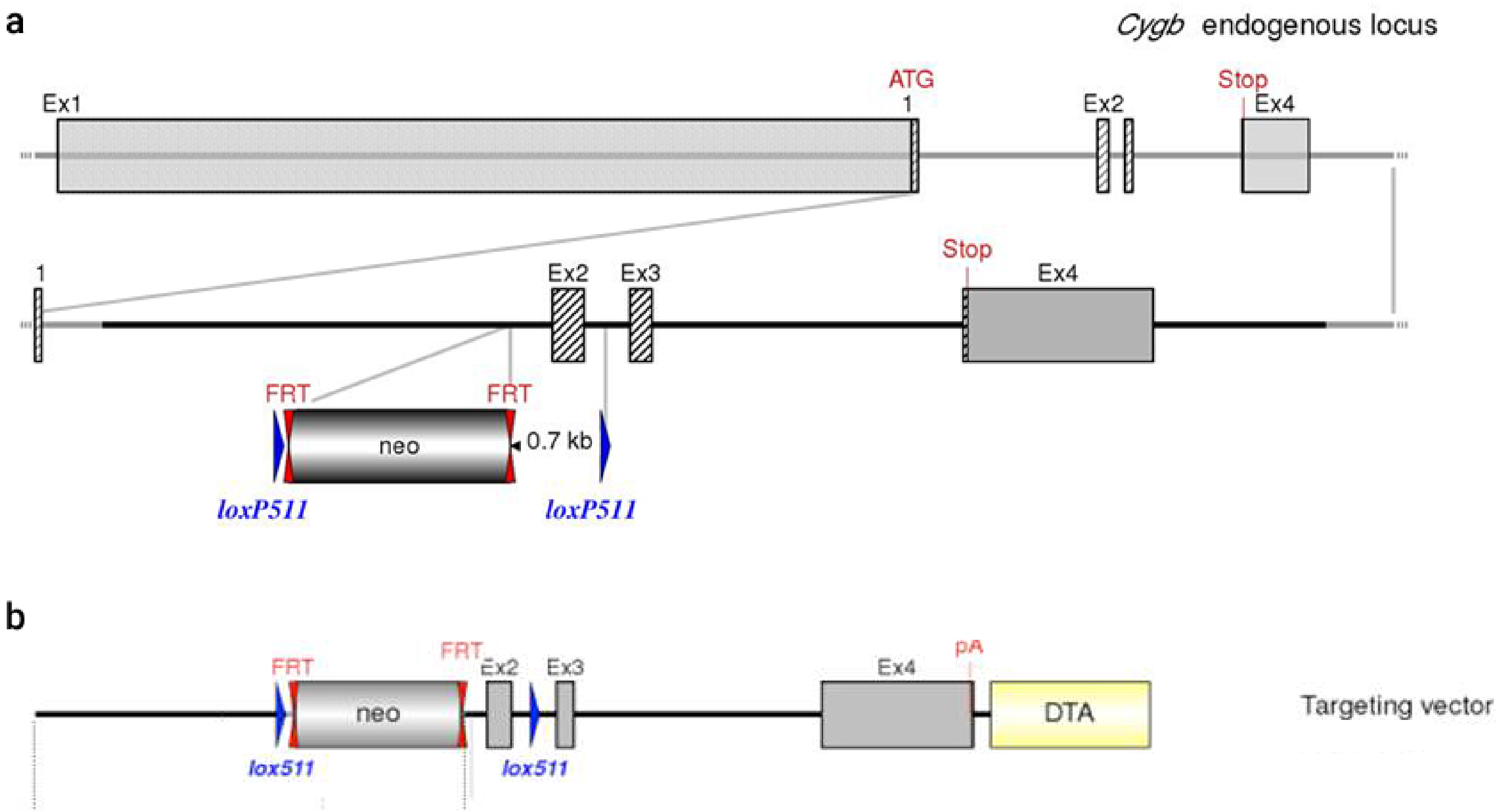

Supplementary figure S1: a) Schematic representation of targeting strategy selected. Diagram is not depicted to scale. Hatched rectangles represent Cygb coding sequences, grey rectangles indicate non-coding exon portions, solid lines represent chromosome sequences. The neomycine positive selection cassette is indicated. Mutated loxP511 sites are represented by blue triangles and FRT sites by double red triangles. The initiation (ATG) and Stop (Stop) codons are indicated. The size of the flanked Cygb sequence to be deleted is specified. **b) Schematic representation of the targeting and positive control vectors.** Diagram is not depicted to scale. loxP sites are represented by blue triangles and FRT sites by double red triangles. Neo: neomycin positive selection cassette; DTA: Diphtheria toxin negative selection cassette. The green portion of the positive control vector represents the extended portion of the homology arm that is not present in the targeting vector.

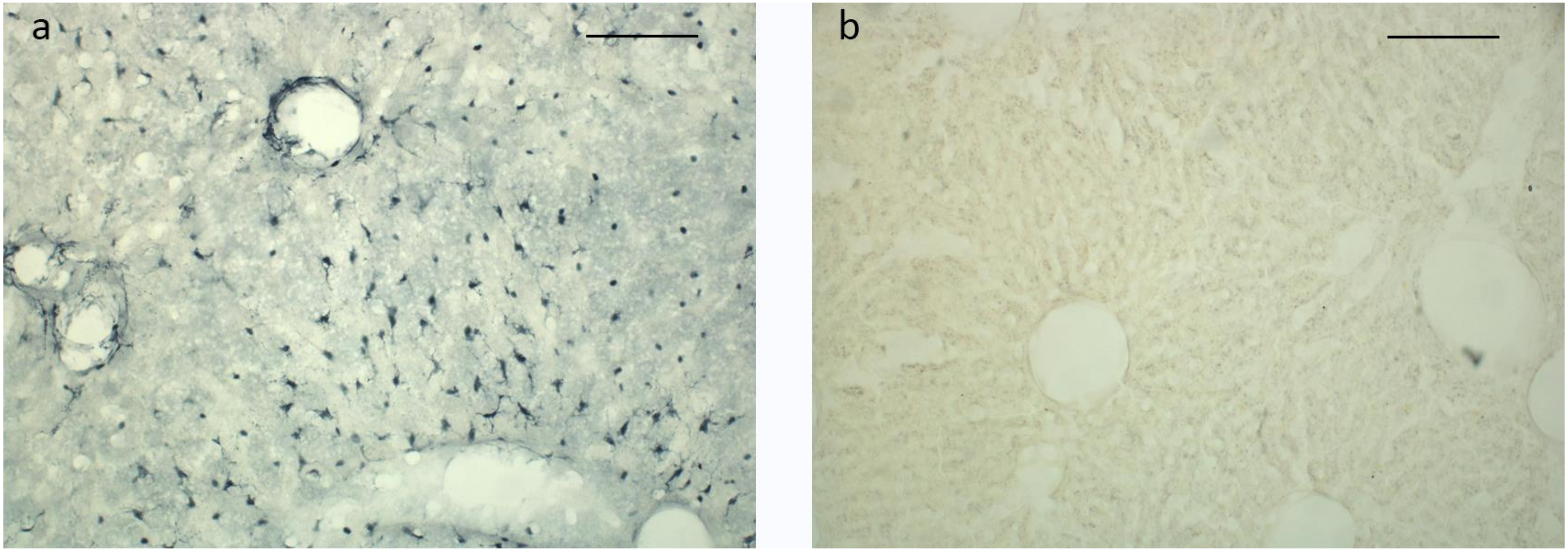

Supplementary figure S2: Liver staining with rabbit anti-human cytoglobin antibody. **a)** WT **b)** Cygb $^{-/-}$

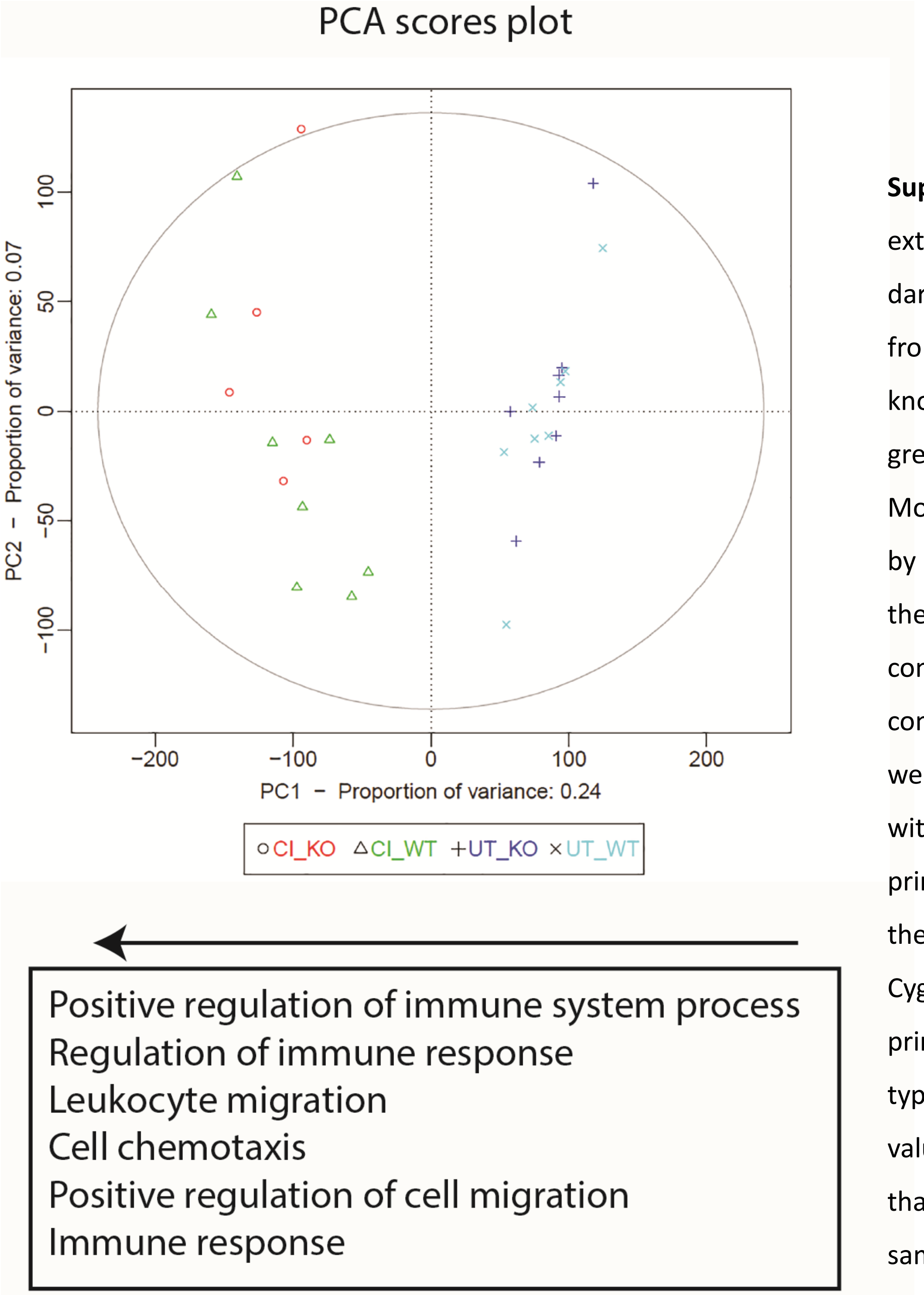

Supplementary figure S3: PCA score plot of colonic samples. RNA was extracted from untreated colons (untreated Cygb -/- knockout: UT_KO, dark blue crosses; untreated wild type: UT_WT, light blue crosses) or from DSS treated, chronically inflamed colons (chronic inflammation knock out: CI_KO, red circles; chronic inflammation wild type: CI_WT, green triangles) and analyzed by microarray analysis (Affymetrix Mouse Gene 2.0 ST array). Unsupervised multivariate analysis followed by GO and promoter overrepresentation analysis was conducted using the pcaGoPromoter package. The score plot of the first two principal components is shown. The variation represented by the first principal component (the X-axis) is correlated with the chronic inflammation (7 weeks, second DSS cycle). The most important GO terms associated with the negative direction (the "inflammation direction") of the first principal component axis is shown. In addition it can be seen than there is a tendency of the positioning of the inflamed samples from Cygb -/- mice (red circles) towards positive values along the second principal component axis whereas the inflamed samples from the wild type mice (green triangles) are mostly positioned towards negative values on the second principal component. Moreover it can be seen that there is no overall difference in the positioning of the untreated samples along either of the two axes.

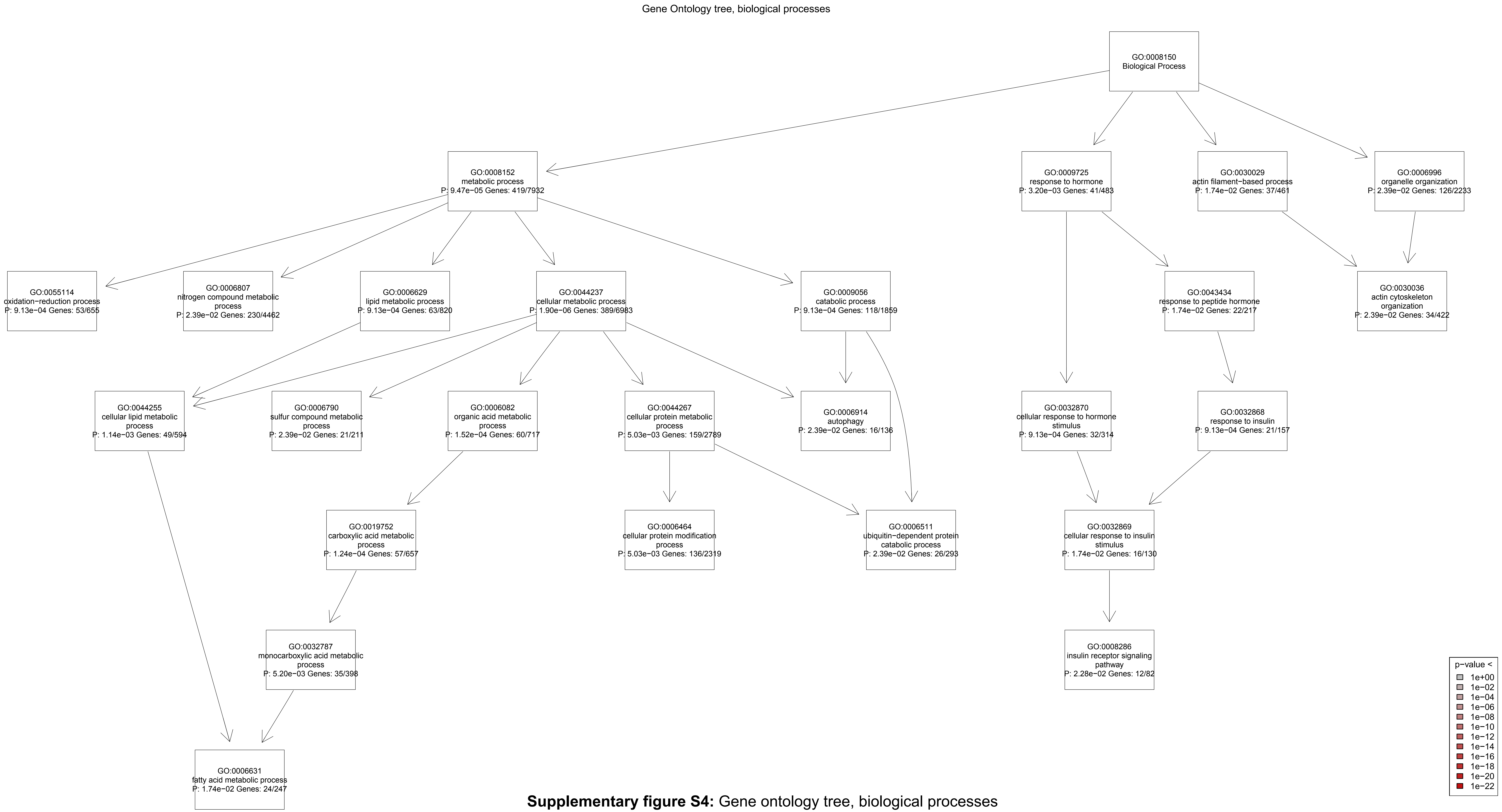

Supplementary figure S4: Gene ontology tree, biological processes

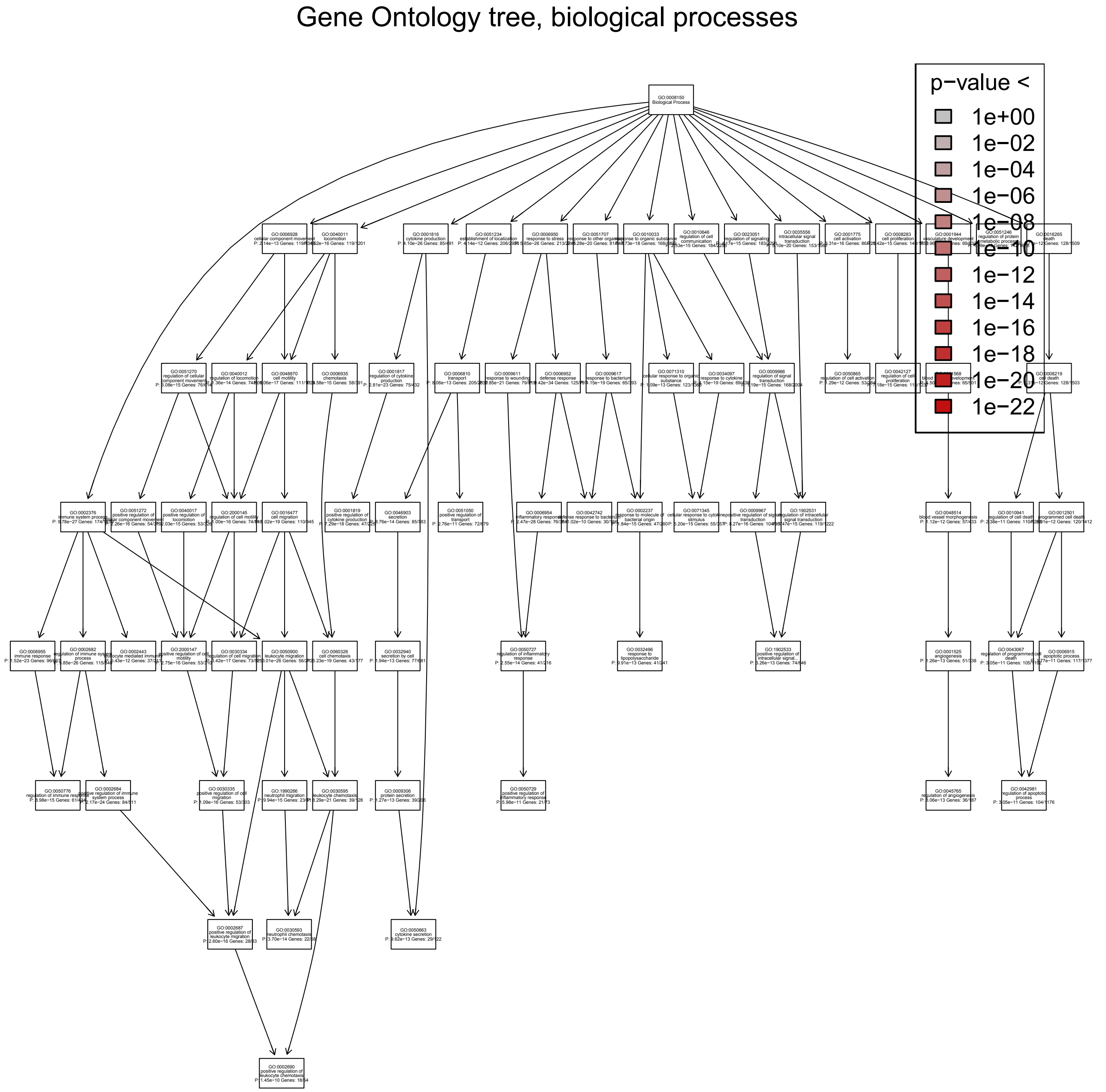

Supplementary figure S5: Gene ontology tree, biological processes

Supplementary tables S1.1-S1.4

Table S1.1

Affy probe ID	Symbol	Gene Name	Fold difference WT/KO	fdr
**17516978**	Nxpe4	neurexophilin and PC-esterase domain family, member 4	16,94509406	5,68E-10
**17272461**	Cygb	cytoglobin	6,577840031	1,34E-08
**17550370**	AI842136	expressed sequence AI842136	1,589520638	9,07E-05
**17300177**	Trav7d-4	T cell receptor alpha variable 7D-4	2,79070594	0,000123205
**17433328**	Per3	period circadian clock 3	2,228560366	0,0001763
**17526550**	AI842136	expressed sequence AI842136	1,685886959	0,0001763
**17298823**	Gm3219	B-cell CLL/lymphoma 7C pseudogene	1,704650801	0,000248343
**17299559**	Pnp2	purine-nucleoside phosphorylase 2	4,003242477	0,000451
**17407934**	Ciart	circadian associated repressor of transcription	1,942646942	0,0004551
**17525329**	St3gal4	ST3 beta-galactoside alpha-2,3-sialyltransferase 4	5,761541311	0,0004551
**17388389**	Cry2	cryptochrome 2 (photolyase-like)	1,681268656	0,000838174
**17313376**	Tef	thyrotroph embryonic factor	1,932710909	0,001190441
**17477979**	Dbp	D site albumin promoter binding protein	2,689878454	0,001190441
**17526149**	Pdzd3	PDZ domain containing 3	1,983234362	0,001190441
**17305005**	Npy4r	neuropeptide Y receptor Y4	1,815853961	0,001820323
**17303625**	Nr1d2	nuclear receptor subfamily 1, group D, member 2	1,623629955	0,00233167
**17526663**	Nxpe2	neurexophilin and PC-esterase domain family, member 2	5,789702639	0,00233167
**17238722**	H60c	histocompatibility 60c	2,451456338	0,003519358
**17339395**	Myom1	myomesin 1	1,617153972	0,003839827
**17267702**	Hlf	hepatic leukemia factor	1,666353	0,004611649
**17301147**	Fam124a	family with sequence similarity 124, member A	1,591939453	0,004611649
**17258653**	Mettl23	methyltransferase like 23	1,52021307	0,004634351
**17515871**	4930581F22Rik	RIKEN cDNA 4930581F22 gene	1,783948796	0,004634351
**17235730**	Smim24	small integral membrane protein 24	1,667586342	0,004833716
**17287148**	Ecm2	extracellular matrix protein 2, female organ and adipocyte specific	1,532319998	0,004897824
**17268955**	Tns4	tensin 4	1,56542669	0,005462207
**17425701**	Mir3095	microRNA 3095	1,694146106	0,005462207
**17472760**	Bhlhe41	basic helix-loop-helix family, member e41	1,536202546	0,005462207
**17547719**	Ahnak2	AHNAK nucleoprotein 2	1,61331611	0,005462207
**17450838**	Rnf212	ring finger protein 212	1,685914334	0,005476638
**17348833**	Ttr	transthyretin	1,527105907	0,006513501
**17526717**	Htr3a	5-hydroxytryptamine (serotonin) receptor 3A	1,615107338	0,006513501

**17334166**	NA	NA	1,648297862	0,00707798
**17421875**	Slc2a5	solute carrier family 2 (facilitated glucose transporter), member 5	1,717350657	0,00707798
**17277760**	5430427M07Rik	RIKEN cDNA 5430427M07 gene	2,294964069	0,007807635
**17468417**	Cml3	camello-like 3	1,888484766	0,007807635
**17364403**	Cyp2c67	cytochrome P450, family 2, subfamily c, polypeptide 67	2,02078486	0,008141636
**17271350**	Abca8a	ATP-binding cassette, sub-family A (ABC1), member 8a	1,822899485	0,00817673
**17319707**	Cyp2d26	cytochrome P450, family 2, subfamily d, polypeptide 26	1,531286817	0,00817673
**17400162**	Selenbp1	selenium binding protein 1	2,073763982	0,00817673
**17334956**	Crebrf	CREB3 regulatory factor	1,580289065	0,01028012
**17400048**	Rorc	RAR-related orphan receptor gamma	1,524210509	0,010944321
**17376124**	Slc20a1	solute carrier family 20, member 1	1,516610881	0,012185261
**17404122**	Slc10a5	solute carrier family 10 (sodium/bile acid cotransporter family), member 5	1,707826652	0,012948381
**17333493**	9030025P20Rik	RIKEN cDNA 9030025P20 gene	1,664410306	0,013383774
**17423577**	Atp6v0d2	ATPase, H+ transporting, lysosomal V0 subunit D2	1,52000713	0,013532909
**17383588**	Ccbl1	cysteine conjugate-beta lyase 1	1,564785103	0,014016393
**17448001**	Ppargc1a	peroxisome proliferative activated receptor, gamma, coactivator 1 alpha	1,528611968	0,014966471
**17400124**	Selenbp2	selenium binding protein 2	1,585449714	0,015159537
**17454193**	Cyp3a13	cytochrome P450, family 3, subfamily a, polypeptide 13	1,659550249	0,015159537
**17252668**	Trpv3	transient receptor potential cation channel, subfamily V, member 3	1,66686476	0,015998317
**17257150**	C130046K22Rik	RIKEN cDNA C130046K22 gene	1,505025491	0,016085182
**17339876**	Qpct	glutaminyl-peptide cyclotransferase (glutaminyl cyclase)	1,66382735	0,016512386
**17376508**	Mir103-2	microRNA 103-2	1,608819739	0,017572477
**17317266**	Fbxo32	F-box protein 32	1,791010144	0,01836186
**17364414**	Cyp2c68	cytochrome P450, family 2, subfamily c, polypeptide 68	1,815403766	0,01949451
**17402113**	Dpyd	dihydropyrimidine dehydrogenase	1,664307901	0,01950064
**17433975**	Mir200b	microRNA 200b	2,071228155	0,01950064
**17517215**	2010007H06Rik	RIKEN cDNA 2010007H06 gene	1,513626785	0,01950064
**17289009**	Hapln1	hyaluronan and proteoglycan link protein 1	1,779609706	0,020785092
**17229036**	Fmo2	flavin containing monooxygenase 2	1,785709	0,02271485
**17399404**	Gm15998	predicted gene 15998	2,714793596	0,022742979
**17525578**	Slc37a2	solute carrier family 37 (glycerol-3-phosphate transporter), member 2	2,287317833	0,022742979
**17274558**	Mboat2	membrane bound O-acyltransferase domain containing 2	1,787370936	0,025409063

**17253215**	Slc6a4	solute carrier family 6 (neurotransmitter transporter, serotonin), member 4	1,585289315	0,025442648
**17274813**	Slc26a3	solute carrier family 26, member 3	2,127228524	0,028650257
**17381589**	Itih2	inter-alpha trypsin inhibitor, heavy chain 2	1,50347836	0,028650257
**17475498**	Cyp2f2	cytochrome P450, family 2, subfamily f, polypeptide 2	2,352114135	0,028650257
**17516762**	Tmprss13	transmembrane protease, serine 13	1,642421575	0,028650257
**17292327**	Mirlet7f-1	microRNA let7f-1	1,593708721	0,030269694
**17231600**	Mir5104	microRNA 5104	1,629361394	0,030483373
**17430227**	Gjb5	gap junction protein, beta 5	1,601738316	0,031514587
**17336526**	Btnl1	butyrophilin-like 1	1,723863465	0,036025407
**17396162**	Car2	carbonic anhydrase 2	1,579834018	0,036025407
**17403058**	Adh7	alcohol dehydrogenase 7 (class IV), mu or sigma polypeptide	2,152534339	0,036085607
**17337580**	Olfr111	olfactory receptor 111	1,605128216	0,036476172
**17313599**	Cyp2d12	cytochrome P450, family 2, subfamily d, polypeptide 12	1,932682486	0,037647047
**17432976**	Angptl7	angiopoietin-like 7	2,00318089	0,037647047
**17317458**	Gsdmc3	gasdermin C3	1,619322797	0,042481643
**17212286**	Slc9a2	solute carrier family 9 (sodium/hydrogen exchanger), member 2	1,522393353	0,046768567
**17306314**	Olfr1512	olfactory receptor 1512	1,54058502	0,049231941
**17364872**	Pyroxd2	pyridine nucleotide-disulphide oxidoreductase domain 2	1,559253782	0,049550673

Table S1.1: List of genes with higher expression in the inflamed colon from wild type mice compared to Cygb $^{-/-}$ mice.

Criteria: Mean fold difference > 1.5 and fdr from t-test <0.05

Inflamed samples (week 7, 2xDSS cycle, chronic inflammation)

Table S1.2

Affy probe ID	Symbol	Gene Name	Fold difference KO/WT	fdr
**17491378**	E2f8	E2F transcription factor 8	1,634243565	0,000546
**17525845**	4931429I11Rik	RIKEN cDNA 4931429I11 gene	1,555221919	0,00157
**17305980**	Ccnb1ip1	cyclin B1 interacting protein 1	2,927387725	0,00157
**17517723**	Rpp25	ribonuclease P/MRP 25 subunit	1,506120785	0,00157
**17258644**	BC018473	cDNA sequence BC018473	23,04484317	0,002056
**17285863**	Hist1h2bb	histone cluster 1, H2bb	1,521549721	0,002679
**17298364**	Nt5dc2	5'-nucleotidase domain containing 2	1,549354238	0,003193
**17511748**	Ces1g	carboxylesterase 1G	1,801067275	0,004897
**17508609**	Nrg1	neuregulin 1	1,566098437	0,005839
**17414802**	Pappa	pregnancy-associated plasma protein A	1,64148824	0,007107
**17294302**	Trip13	thyroid hormone receptor interactor 13	1,55267672	0,008198
**17390810**	Gatm	glycine amidinotransferase (L-arginine:glycine amidinotransferase)	1,735121386	0,008326
**17481960**	Arntl	aryl hydrocarbon receptor nuclear translocator-like	1,707390014	0,00851
**17503023**	Asf1b	anti-silencing function 1B histone chaperone	1,541986817	0,00851
**17532334**	Cck	cholecystokinin	1,679692197	0,00851
**17429454**	Ctps	cytidine 5'-triphosphate synthase	1,599626302	0,00851
**17291005**	Hist1h1b	histone cluster 1, H1b	1,645566233	0,00851
**17428553**	Rad54l	RAD54 like (S. cerevisiae)	1,565738507	0,00851
**17431720**	Alpl	alkaline phosphatase, liver/bone/kidney	1,521498132	0,009426
**17457887**	Tmem139	transmembrane protein 139	1,571646894	0,010597
**17331828**	Cldn8	claudin 8	1,601281977	0,012949
**17338371**	Trem3	triggering receptor expressed on myeloid cells 3	1,657092808	0,013117
**17470031**	Alox5	arachidonate 5-lipoxygenase	1,628847778	0,013299
**17379606**	Mmp9	matrix metallopeptidase 9	2,107634246	0,013299
**17502573**	Hmox1	heme oxygenase (decycling) 1	1,669209815	0,014911
**17418141**	Bmp8b	bone morphogenetic protein 8b	1,712093992	0,017181
**17459335**	Igkv14-111	immunoglobulin kappa variable 14-111	2,010126991	0,017181
**17473248**	Ttyh1	tweety homolog 1 (Drosophila)	1,673137594	0,019222
**17385757**	Gcg	glucagon	1,966633335	0,020872
**17513135**	Ldhd	lactate dehydrogenase D	1,532459495	0,020882
**17219248**	Adamts4	a disintegrin-like and metallopeptidase (reprolysin type) with thrombospondin type 1 motif, 4	1,704614508	0,021867

**17514541**	Mmp10	matrix metallopeptidase 10	2,384620058	0,021867
**17533713**	Timp1	tissue inhibitor of metalloproteinase 1	1,915117509	0,022819
**17266952**	Ccl9	chemokine (C-C motif) ligand 9	1,530465106	0,024195
**17353957**	Spry4	sprouty homolog 4 (Drosophila)	1,586817726	0,024195
**17439029**	Areg	amphiregulin	1,661397773	0,024724
**17285742**	Hist1h2bk	histone cluster 1, H2bk	1,645298472	0,024724
**17517812**	Sema7a	sema domain, immunoglobulin domain (Ig), and GPI membrane anchor, (semaphorin) 7A	1,6833067	0,025596
**17266851**	Slfn9	schlafen 9	1,676567826	0,025596
**17526836**	Tex12	testis expressed gene 12	1,71387679	0,025596
**17487533**	Vmn1r103	vomeronasal 1 receptor 103	1,570013448	0,025596
**17291881**	F13a1	coagulation factor XIII, A1 subunit	1,72378076	0,028593
**17292634**	Nfil3	nuclear factor, interleukin 3, regulated	1,599873547	0,03005
**17318794**	Apol7c	apolipoprotein L 7c	1,53065167	0,030819
**17492947**	Cemip	cell migration inducing protein, hyaluronan binding	1,97702046	0,030819
**17408897**	Chil3	chitinase-like 3	3,881711991	0,030819
**17514482**	Mmp13	matrix metallopeptidase 13	2,639327508	0,030819
**17301428**	Pbk	PDZ binding kinase	1,591183106	0,030819
**17491193**	Saa3	serum amyloid A 3	3,0997362	0,030819
**17393789**	Tgm2	transglutaminase 2, C polypeptide	1,638305196	0,030819
**17312716**	Csf2rb	colony stimulating factor 2 receptor, beta, low-affinity (granulocyte-macrophage)	1,513482104	0,031464
**17212211**	Il1rl1	interleukin 1 receptor-like 1	1,804785083	0,031464
**17383892**	Lcn2	lipocalin 2	1,829227313	0,033402
**17276776**	Arg2	arginase type II	1,656653553	0,035984
**17438995**	Cxcl2	chemokine (C-X-C motif) ligand 2	2,76109925	0,035984
**17356427**	Fosl1	fos-like antigen 1	1,57055952	0,035984
**17344508**	Gm9840	predicted gene 9840	1,524263372	0,035984
**17285438**	Inhba	inhibin beta-A	1,696159573	0,035984
**17429632**	Mfsd2a	major facilitator superfamily domain containing 2A	1,960310352	0,035984
**17514515**	Mmp3	matrix metallopeptidase 3	2,754368079	0,035984
**17471550**	Olr1	oxidized low density lipoprotein (lectin-like) receptor 1	2,567688818	0,035984
**17270048**	Ppy	pancreatic polypeptide	1,658197726	0,035984
**17321895**	4732456N10Rik	RIKEN cDNA 4732456N10 gene	1,662515222	0,037635
**17345775**	A530064D06Rik	RIKEN cDNA A530064D06 gene	1,597886589	0,037635

**17337228**	Ier3	immediate early response 3	1,574433224	0,037635
**17278321**	Serpina3m	serine (or cysteine) peptidase inhibitor, clade A, member 3M	2,461105677	0,037635
**17246803**	Osm	oncostatin M	1,839644436	0,039986
**17211369**	Il17a	interleukin 17A	1,613213112	0,040707
**17438987**	Cxcl1	chemokine (C-X-C motif) ligand 1	1,750226049	0,041268
**17481622**	Olfr503	olfactory receptor 503	1,528035676	0,042611
**17218060**	Ptgs2	prostaglandin-endoperoxide synthase 2	2,543337484	0,043099
**17343628**	Angptl4	angiopoietin-like 4	1,717352117	0,048982
**17449718**	Cxcl10	chemokine (C-X-C motif) ligand 10	2,44272561	0,048982
**17439021**	Ereg	epiregulin	2,023200931	0,048982
**17514553**	Mmp8	matrix metallopeptidase 8	3,118206202	0,048982
**17447081**	Spon2	spondin 2, extracellular matrix protein	1,584082944	0,048982
**17280184**	Zfp125	zinc finger protein 125	3,146946326	0,048982
**17278404**	Bdkrb1	bradykinin receptor, beta 1	1,545299927	0,049487
**17518813**	C2cd4b	C2 calcium-dependent domain containing 4B	1,884835305	0,049487
**17474974**	Plaur	plasminogen activator, urokinase receptor	1,792923445	0,049487

Table S1.2: List of genes with higher expression in the inflamed colon from Cygb $^{-/-}$ mice compared to wild type mice.
Criteria: Mean fold difference > 1.5 and fdr from t-test <0.05

Inflamed samples (week 7, 2xDSS cycle, chronic inflammation)

Table S1.3

Affy probe ID	Symbol	Gene Name	Fold difference WT/KO	fdr
**17272461**	Cygb	cytoglobin	6,338146435	2,16E-14
**17258653**	Mettl23	methyltransferase like 23	1,503462927	1,15E-07
**17271033**	Kpna2	karyopherin (importin) alpha 2	1,499638346	3,41E-05
**17309457**	Dzip1	DAZ interacting protein 1	1,533691911	0,001197
**17404337**	Cpa3	carboxypeptidase A3, mast cell	1,720704222	0,001826
**17302606**	Tpm3	tropomyosin 3, gamma	1,686057843	0,001834
**17257150**	C130046K22Rik	RIKEN cDNA C130046K22 gene	2,057878075	0,002741
**17298775**	Anxa8	annexin A8	1,782253607	0,005494
**17477282**	Klk15	kallikrein related-peptidase 15	1,706671811	0,006457
**17284466**	Igh-VJ558	immunoglobulin heavy chain (J558 family)	1,750032252	0,007146
**17489542**	Scgb2b20	secretoglobin, family 2B, member 20	2,015328899	0,015765
**17289787**	Rps3a1	ribosomal protein S3A1	1,519024179	0,027187
**17284582**	Ighv1-54	immunoglobulin heavy variable V1-54	2,150796146	0,034987
**17459344**	Igk	immunoglobulin kappa chain complex	2,209597572	0,040093
**17467430**	Igkv4-72	immunoglobulin kappa chain variable 4-72	1,529554088	0,04977

Table S1.3: List of genes with higher expression in the normal colon from wild type mice compared to Cygb $^{-/-}$ mice.

Table S1.4

Affy probe ID	Symbol	Gene Name	Fold difference WT/KO	fdr
**17258644**	BC018473	cDNA sequence BC018473	5,883278	8,29E-11
**17271486**	Abca5	ATP-binding cassette, sub-family A (ABC1), member 5	1,865528	2,43E-07
**17483098**	Gdpd3	glycerophosphodiester phosphodiesterase domain containing 3	3,609797	0,001052
**17326062**	Retnlb	resistin like beta	5,198812	0,00174
**17449346**	Ugt2b5	UDP glucuronosyltransferase 2 family, polypeptide B5	1,607831	0,001859
**17302289**	Pcdh17	protocadherin 17	2,582879	0,003505
**17330769**	Ahcy	S-adenosylhomocysteine hydrolase	1,626395	0,006967
**17545899**	S100g	S100 calcium binding protein G	1,750887	0,012131
**17407934**	Ciart	circadian associated repressor of transcription	1,840983	0,017545
**17395079**	Zbp1	Z-DNA binding protein 1	1,539852	0,021378
**17284417**	Igh-VJ558	immunoglobulin heavy chain (J558 family)	3,56838	0,035206
**17284474**	Igh-VJ558	immunoglobulin heavy chain (J558 family)	1,495277	0,041078
**17329009**	Iglv1	immunoglobulin lambda variable 1	1,913274	0,047622

Table S1.4: List of genes with higher expression in the normal colon from Cygb $^{-/-}$ mice compared to wild type mice.

Criteria: Mean fold difference > 1.5 and fdr from t-test <0.05

Supplementary tables S2.1-S2.4

Table S2.1

Entrez ID	Symbol	Gene Name	Fold difference WT/KO	p-val
**20249**	Scd1	stearoyl-Coenzyme A desaturase 1	1,356677398	2,56E-05
**11364**	Acadm	acyl-Coenzyme A dehydrogenase, medium chain	1,27646525	0,000561
**56794**	Hacl1	2-hydroxyacyl-CoA lyase 1	1,250941269	0,001487
**231070**	Insig1	insulin induced gene 1	1,355183458	0,004379
**72674**	Adipor1	adiponectin receptor 1	1,072113608	0,006199
**19017**	Ppargc1a	peroxisome proliferative activated receptor, gamma, coactivator 1 alpha	1,528611968	0,008761
**93759**	Sirt1	sirtuin 1	1,16673699	0,011352
**14732**	Gpam	glycerol-3-phosphate acyltransferase, mitochondrial	1,151317792	0,013366
**67452**	Pnpla8	patatin-like phospholipase domain containing 8	1,096135487	0,018151
**108099**	Prkag2	protein kinase, AMP-activated, gamma 2 non-catalytic subunit	1,147805075	0,021596
**230796**	Wdtc1	WD and tetratricopeptide repeats 1	1,17734385	0,025838
**107476**	Acaca	acetyl-Coenzyme A carboxylase alpha	1,166081223	0,027343
**52538**	Acaa2	acetyl-Coenzyme A acyltransferase 2 (mitochondrial 3-oxoacyl-Coenzyme A thiolase)	1,191577305	0,028117
**54486**	Hpgds	hematopoietic prostaglandin D synthase	1,3912346	0,029585
**18604**	Pdk2	pyruvate dehydrogenase kinase, isoenzyme 2	1,35648685	0,031631
**104112**	Acly	ATP citrate lyase	1,164917956	0,039023
**15446**	Hpgd	hydroxyprostaglandin dehydrogenase 15 (NAD)	1,312857141	0,043529
**74147**	Ehhadh	enoyl-Coenzyme A, hydratase/3-hydroxyacyl Coenzyme A dehydrogenase	1,209874683	0,048791
**16367**	Irs1	insulin receptor substrate 1	1,23961086	0,057699
**13614**	Edn1	endothelin 1	1,405299523	0,103405
**66887**	Lonp2	lon peptidase 2, peroxisomal	1,06790438	0,132672
**72674**	Adipor1	adiponectin receptor 1	1,102594597	0,184396
**72674**	Adipor1	adiponectin receptor 1	1,102594597	0,184396
**26457**	Slc27a1	solute carrier family 27 (fatty acid transporter), member 1	1,139809526	0,229577
**18484**	Pam	peptidylglycine alpha-amidating monooxygenase	1,093968741	0,284565
**20397**	Sgpl1	sphingosine phosphate lyase 1	1,041421973	0,364329
**16367**	Irs1	insulin receptor substrate 1	1,039041054	0,688691

Table S2.1: List of genes annotated with the term: GO:006631: Fatty acid metabolic process

Method: The 1200 PCA loadings having the highest values (positive direction) for the second principal component of the PCA of inflamed samples were analyzed for overrepresentation of GO terms using the pcaGoPromoter package (HANSEN, M., GERDS, T. A., NIELSEN, O. H., SEIDELIN, J. B., TROELSEN, J. T. & OLSEN, J. 2012. pcaGoPromoter--an R package for biological and regulatory interpretation of principal components in genome-wide gene expression data. *PLoS One,* 7**,** e32394.)

Table S2.2

Entrez ID	Symbol	Gene Name	Fold difference WT/KO	p-val
**19193**	Pipox	pipecolic acid oxidase	1,354095669	0,003052
**14732**	Gpam	glycerol-3-phosphate acyltransferase, mitochondrial	1,151317792	0,013366
**75475**	Oplah	5-oxoprolinase (ATP-hydrolysing)	1,187552492	0,019513
**107476**	Acaca	acetyl-Coenzyme A carboxylase alpha	1,166081223	0,027343
**52538**	Acaa2	acetyl-Coenzyme A acyltransferase 2 (mitochondrial 3-oxoacyl-Coenzyme A thiolase)	1,191577305	0,028117
**18604**	Pdk2	pyruvate dehydrogenase kinase, isoenzyme 2	1,35648685	0,031631
**104112**	Acly	ATP citrate lyase	1,164917956	0,039023
**17850**	Mut	methylmalonyl-Coenzyme A mutase	1,130843697	0,040212
**74147**	Ehhadh	enoyl-Coenzyme A, hydratase/3-hydroxyacyl Coenzyme A dehydrogenase	1,209874683	0,048791
**83429**	Ctns	cystinosis, nephropathic	1,193937492	0,050756
**108645**	Mat2b	methionine adenosyltransferase II, beta	1,089483231	0,051629
**20887**	Sult1a1	sulfotransferase family 1A, phenol-preferring, member 1	1,611684377	0,054045
**14862**	Gstm1	glutathione S-transferase, mu 1	1,166648273	0,060722
**54200**	Sult2b1	sulfotransferase family, cytosolic, 2B, member 1	1,229863358	0,060725
**23971**	Papss1	3'-phosphoadenosine 5'-phosphosulfate synthase 1	1,299918764	0,062925
**18260**	Ocln	occludin	1,128591833	0,063902
**23971**	Papss1	3'-phosphoadenosine 5'-phosphosulfate synthase 1	1,106767328	0,088231
**23971**	Papss1	3'-phosphoadenosine 5'-phosphosulfate synthase 1	1,106767328	0,088231
**269378**	Ahcy	S-adenosylhomocysteine hydrolase	0,652329612	0,105119
**232223**	Txnrd3	thioredoxin reductase 3	1,154347237	0,110684
**269378**	Ahcy	S-adenosylhomocysteine hydrolase	1,167365783	0,123049
**246277**	Csad	cysteine sulfinic acid decarboxylase	1,139956551	0,136897
**18597**	Pdha1	pyruvate dehydrogenase E1 alpha 1	1,098606027	0,144881
**14854**	Gss	glutathione synthetase	1,066026108	0,211398
**17850**	Mut	methylmalonyl-Coenzyme A mutase	1,063252536	0,32835

Table S2.2: List of genes annotated with the term: GO:006790: Sulfur compound metabolic process

Method: The 1200 PCA loadings having the highest values (positive direction) for the second principal component of the PCA of inflamed samples were analyzed for overrepresentation of GO terms using the pcaGoPromoter package (HANSEN, M., GERDS, T. A., NIELSEN, O. H., SEIDELIN, J. B., TROELSEN, J. T. & OLSEN, J. 2012. pcaGoPromoter--an R package for biological and regulatory interpretation of principal components in genome-wide gene expression data. *PLoS One,* 7**,** e32394.)

Table S2.3

Entrez ID	Symbol	Gene Name	Fold difference WT/KO	p-val
**16924**	Lnx1	ligand of numb-protein X 1	1,414711	0,001092
**218203**	Mylip	myosin regulatory light chain interacting protein	1,420797	0,001212
**50789**	Fbxl3	F-box and leucine-rich repeat protein 3	1,317114	0,002682
**67490**	Ufl1	UFM1 specific ligase 1	1,126042	0,002921
**76454**	Fbxo31	F-box protein 31	1,165943	0,004064
**230249**	AI314180	expressed sequence AI314180	1,15287	0,004566
**16396**	Itch	itchy, E3 ubiquitin protein ligase	1,140556	0,008782
**93759**	Sirt1	sirtuin 1	1,166737	0,011352
**66894**	Wwp2	WW domain containing E3 ubiquitin protein ligase 2	1,293351	0,01455
**58799**	Crbn	cereblon	1,140585	0,017692
**74132**	Rnf6	ring finger protein (C3H2C3 type) 6	1,069568	0,026321
**22644**	Rnf103	ring finger protein 103	1,189097	0,02952
**68031**	Rnf146	ring finger protein 146	1,116345	0,042724
**68795**	Ubr3	ubiquitin protein ligase E3 component n-recognin 3	1,10537	0,044734
**15204**	Herc2	hect (homologous to the E6-AP (UBE3A) carboxyl terminus) domain and RCC1 (CHC1)-like domain (RLD) 2	1,124498	0,046583
**76857**	Spopl	speckle-type POZ protein-like	1,142693	0,059084
**75744**	Svip	small VCP/p97-interacting protein	1,129542	0,067909
**71472**	Usp19	ubiquitin specific peptidase 19	1,0874	0,085242
**26374**	Rfwd2	ring finger and WD repeat domain 2	1,086283	0,092205
**50753**	Fbxo8	F-box protein 8	1,087771	0,092805
**360216**	Zranb1	zinc finger, RAN-binding domain containing 1	1,161362	0,105411
**231600**	Chfr	checkpoint with forkhead and ring finger domains	1,09196	0,213313
**231600**	Chfr	checkpoint with forkhead and ring finger domains	1,09196	0,213313
**15204**	Herc2	hect (homologous to the E6-AP (UBE3A) carboxyl terminus) domain and RCC1 (CHC1)-like domain (RLD) 2	1,093863	0,218153
**11907**	Ate1	arginyltransferase 1	1,050392	0,240746
**99375**	Cul4a	cullin 4A	1,05531	0,283898
**14198**	Fhit	fragile histidine triad gene	1,106432	0,319896
**107568**	Wwp1	WW domain containing E3 ubiquitin protein ligase 1	1,038338	0,481989

**231600**	Chfr	checkpoint with forkhead and ring finger domains	0,991564	0,680829

Table S2.3: List of genes annotated with the term: GO:006511: ubiquitin-dependent protein catabolic process

Method: The 1200 PCA loadings having the highest values (positive direction) for the second principal component of the PCA of inflamed samples were analyzed for overrepresentation of GO terms using the pcaGoPromoter package (HANSEN, M., GERDS, T. A., NIELSEN, O. H., SEIDELIN, J. B., TROELSEN, J. T. & OLSEN, J. 2012. pcaGoPromoter--an R package for biological and regulatory interpretation of principal components in genome-wide gene expression data. *PLoS One,* 7**,** e32394.)

Table S2.4

Entrez ID	Symbol	Gene Name	Fold difference WT/KO	p-val
**108155**	Ogt	O-linked N-acetylglucosamine (GlcNAc) transferase (UDP-N-acetylglucosamine:polypeptide-N-acetylglucosaminyl transferase)	1,210525	0,001346
**19062**	Inpp5k	inositol polyphosphate 5-phosphatase K	1,238488	0,00187
**72674**	Adipor1	adiponectin receptor 1	1,072114	0,006199
**50915**	Grb14	growth factor receptor bound protein 14	1,214968	0,006986
**16337**	Insr	insulin receptor	1,170264	0,007071
**18762**	Prkcz	protein kinase C, zeta	1,207984	0,009475
**93759**	Sirt1	sirtuin 1	1,166737	0,011352
**18604**	Pdk2	pyruvate dehydrogenase kinase, isoenzyme 2	1,356487	0,031631
**16367**	Irs1	insulin receptor substrate 1	1,239611	0,057699
**54601**	Foxo4	forkhead box O4	1,156806	0,067306
**107351**	Kank1	KN motif and ankyrin repeat domains 1	1,091611	0,084997
**18710**	Pik3r3	phosphatidylinositol 3 kinase, regulatory subunit, polypeptide 3 (p55)	1,389881	0,114489
**72674**	Adipor1	adiponectin receptor 1	1,102595	0,184396
**72674**	Adipor1	adiponectin receptor 1	1,102595	0,184396
**19062**	Inpp5k	inositol polyphosphate 5-phosphatase K	1,078192	0,318703
**16367**	Irs1	insulin receptor substrate 1	1,039041	0,688691

Table S2.4: List of genes annotated with the term: GO:008286: insulin receptor signaling pathway

Method: The 1200 PCA loadings having the highest values (positive direction) for the second principal component of the PCA of inflamed samples were analyzed for overrepresentation of GO terms using the pcaGoPromoter package (HANSEN, M., GERDS, T. A., NIELSEN, O. H., SEIDELIN, J. B., TROELSEN, J. T. & OLSEN, J. 2012. pcaGoPromoter--an R package for biological and regulatory interpretation of principal components in genome-wide gene expression data. *PLoS One,* 7**,** e32394.)

Supplementary tables S3.1-S3.7

Table S3.1

Entrez ID	Symbol	Gene Name	Fold difference KO/WT	p-val
**12317**	Calr	calreticulin	1,143642222	0,003689547
**117149**	Tirap	toll-interleukin 1 receptor (TIR) domain-containing adaptor protein	1,199439967	0,005892
**21825**	Thbs1	thrombospondin 1	1,437723299	0,016174791
**15945**	Cxcl10	chemokine (C-X-C motif) ligand 10	2,44272561	0,044803522
**12317**	Calr	calreticulin	1,184642546	0,059315132
**27226**	Pla2g7	phospholipase A2, group VII (platelet-activating factor acetylhydrolase, plasma)	1,436076827	0,060273925
**16176**	Il1b	interleukin 1 beta	1,905150026	0,084803163
**20303**	Ccl4	chemokine (C-C motif) ligand 4	1,806492031	0,098503362
**20296**	Ccl2	chemokine (C-C motif) ligand 2	1,864354159	0,099166109
**18787**	Serpine1	serine (or cysteine) peptidase inhibitor, clade E, member 1	1,556913819	0,100627551
**16803**	Lbp	lipopolysaccharide binding protein	1,383979537	0,116070176
**12765**	Cxcr2	chemokine (C-X-C motif) receptor 2	1,63756528	0,140666222
**11491**	Adam17	a disintegrin and metallopeptidase domain 17	1,058605094	0,147737413
**12765**	Cxcr2	chemokine (C-X-C motif) receptor 2	1,483750117	0,148694703
**50930**	Tnfsf14	tumor necrosis factor (ligand) superfamily, member 14	1,220854404	0,175822432
**20343**	Sell	selectin, lymphocyte	1,503068041	0,181181171
**12768**	Ccr1	chemokine (C-C motif) receptor 1	1,452901228	0,183596858
**22339**	Vegfa	vascular endothelial growth factor A	1,197489798	0,275979508
**12772**	Ccr2	chemokine (C-C motif) receptor 2	1,362893367	0,277259703
**12317**	Calr	calreticulin	1,094774667	0,321448205
**14747**	Cmklr1	chemokine-like receptor 1	1,083523427	0,55670659

Table S3.1: List of genes annotated with the term: GO:002690: Positive regulation of leukocyte chemotaxis

Method: The 1200 PCA loadings having the lowest values (negative direction) for the second principal component of the PCA of inflamed samples were analyzed for overrepresentation of GO terms using the pcaGoPromoter package (HANSEN, M., GERDS, T. A., NIELSEN, O. H., SEIDELIN, J. B., TROELSEN, J. T. & OLSEN, J. 2012. pcaGoPromoter--an R package for biological and regulatory interpretation of principal components in genome-wide gene expression data. *PLoS One,* 7**,** e32394.)

Table S3.2

Entrez ID	Symbol	Gene Name	Fold difference KO/WT	p-val
**58218**	Trem3	triggering receptor expressed on myeloid cells 3	1,657092808	0,003607
**268973**	Nlrc4	NLR family, CARD domain containing 4	1,231243659	0,026239
**19720**	Trim27	tripartite motif-containing 27	1,077340255	0,047614
**21942**	Tnfrsf9	tumor necrosis factor receptor superfamily, member 9	1,497761155	0,054009
**58217**	Trem1	triggering receptor expressed on myeloid cells 1	1,890782778	0,07476
**12475**	Cd14	CD14 antigen	1,473474488	0,080085
**56619**	Clec4e	C-type lectin domain family 4, member e	2,530125387	0,081897
**216799**	Nlrp3	NLR family, pyrin domain containing 3	1,919111518	0,08831
**20302**	Ccl3	chemokine (C-C motif) ligand 3	2,70627181	0,090362
**16175**	Il1a	interleukin 1 alpha	2,585228093	0,09377
**17319**	Mif	macrophage migration inhibitory factor	1,149761944	0,094491
**12654**	Chil1	chitinase-like 1	1,764722082	0,097187
**56620**	Clec4n	C-type lectin domain family 4, member n	1,322179181	0,106317
**16190**	Il4ra	interleukin 4 receptor, alpha	1,290700668	0,11366
**16193**	Il6	interleukin 6	2,014699865	0,128143
**21926**	Tnf	tumor necrosis factor	1,355574202	0,186093
**15978**	Ifng	interferon gamma	1,466395008	0,201587
**12978**	Csf1r	colony stimulating factor 1 receptor	1,187319041	0,2133
**19073**	Srgn	serglycin	1,194069244	0,219673
**12774**	Ccr5	chemokine (C-C motif) receptor 5	1,384260071	0,241965
**14462**	Gata3	GATA binding protein 3	1,104580722	0,272671
**24088**	Tlr2	toll-like receptor 2	1,122169952	0,280115
**60533**	Cd274	CD274 antigen	1,457233851	0,290625
**23845**	Clec5a	C-type lectin domain family 5, member a	1,324283295	0,291246
**140497**	AF251705	cDNA sequence AF251705	1,177412867	0,293763
**14191**	Fgr	Gardner-Rasheed feline sarcoma viral (Fgr) oncogene homolog	1,303455198	0,328007

**16822**	Lcp2	lymphocyte cytosolic protein 2	1,212943926	0,339547
**75234**	Rnf19b	ring finger protein 19B	1,095964969	0,488414
**257632**	Nod2	nucleotide-binding oligomerization domain containing 2	0,952541955	0,517038
**19720**	Trim27	tripartite motif-containing 27	0,958184754	0,544742
**257632**	Nod2	nucleotide-binding oligomerization domain containing 2	1,077709849	0,587887

Table S3.2: List of genes annotated with the term: GO:0050663: Cytokine secretion

Method: The 1200 PCA loadings having the lowest values (negative direction) for the second principal component of the PCA of inflamed samples were analyzed for overrepresentation of GO terms using the pcaGoPromoter package (HANSEN, M., GERDS, T. A., NIELSEN, O. H., SEIDELIN, J. B., TROELSEN, J. T. & OLSEN, J. 2012. pcaGoPromoter--an R package for biological and regulatory interpretation of principal components in genome-wide gene expression data. *PLoS One,* 7**,** e32394.)

Table S3.3

Entrez ID	Symbol	Gene Name	Fold difference KO/WT	p-val
**21817**	Tgm2	transglutaminase 2, C polypeptide	1,638305	0,018782
**19225**	Ptgs2	prostaglandin-endoperoxide synthase 2	2,543337	0,03825
**11541**	Adora2b	adenosine A2b receptor	1,27513	0,042341
**13649**	Egfr	epidermal growth factor receptor	1,149276	0,063258
**16176**	Il1b	interleukin 1 beta	1,90515	0,084803
**20302**	Ccl3	chemokine (C-C motif) ligand 3	2,706272	0,090362
**17319**	Mif	macrophage migration inhibitory factor	1,149762	0,094491
**11690**	Alox5ap	arachidonate 5-lipoxygenase activating protein	1,65731	0,099824
**18787**	Serpine1	serine (or cysteine) peptidase inhibitor, clade E, member 1	1,556914	0,100628
**20202**	S100a9	S100 calcium binding protein A9 (calgranulin B)	1,858873	0,115158
**16803**	Lbp	lipopolysaccharide binding protein	1,38398	0,11607
**20201**	S100a8	S100 calcium binding protein A8 (calgranulin A)	2,185588	0,119307
**16193**	Il6	interleukin 6	2,0147	0,128143
**14127**	Fcer1g	Fc receptor, IgE, high affinity I, gamma polypeptide	1,330686	0,14568
**14131**	Fcgr3	Fc receptor, IgG, low affinity III	1,376853	0,16662
**21926**	Tnf	tumor necrosis factor	1,355574	0,186093
**14129**	Fcgr1	Fc receptor, IgG, high affinity I	1,377935	0,239132
**12774**	Ccr5	chemokine (C-C motif) receptor 5	1,38426	0,241965
**20851**	Stat5b	signal transducer and activator of transcription 5B	1,097249	0,269314
**12772**	Ccr2	chemokine (C-C motif) receptor 2	1,362893	0,27726
**21943**	Tnfsf11	tumor necrosis factor (ligand) superfamily, member 11	1,172481	0,347524
**11541**	Adora2b	adenosine A2b receptor	1,030538	0,63402

Table S3.3: List of genes annotated with the term: GO:0050729: Positive regulation of Inflammatory response

Method: The 1200 PCA loadings having the lowest values (negative direction) for the second principal component of the PCA of inflamed samples were analyzed for overrepresentation of GO terms using the pcaGoPromoter package (HANSEN, M., GERDS, T. A., NIELSEN, O. H., SEIDELIN, J. B., TROELSEN, J. T. & OLSEN, J. 2012. pcaGoPromoter--an R package for biological and regulatory interpretation of principal components in genome-wide gene expression data. *PLoS One,* 7**,** e32394.)

Table S3.4

Entrez ID	Symbol	Gene Name	Fold difference KO/WT	p-val
**117149**	Tirap	toll-interleukin 1 receptor (TIR) domain-containing adaptor protein	1,19944	0,005892
**723839**	Mir323	microRNA 323	1,217001	0,009882
**19401**	Rara	retinoic acid receptor, alpha	1,128443	0,019123
**21938**	Tnfrsf1b	tumor necrosis factor receptor superfamily, member 1b	1,336643	0,02436
**19225**	Ptgs2	prostaglandin-endoperoxide synthase 2	2,543337	0,03825
**15945**	Cxcl10	chemokine (C-X-C motif) ligand 10	2,442726	0,044804
**12061**	Bdkrb1	bradykinin receptor, beta 1	1,5453	0,049071
**12608**	Cebpb	CCAAT/enhancer binding protein (C/EBP), beta	1,406472	0,056195
**12475**	Cd14	CD14 antigen	1,473474	0,080085
**16176**	Il1b	interleukin 1 beta	1,90515	0,084803
**216799**	Nlrp3	NLR family, pyrin domain containing 3	1,919112	0,08831
**11535**	Adm	adrenomedullin	1,58963	0,091321
**17319**	Mif	macrophage migration inhibitory factor	1,149762	0,094491
**20296**	Ccl2	chemokine (C-C motif) ligand 2	1,864354	0,099166
**18787**	Serpine1	serine (or cysteine) peptidase inhibitor, clade E, member 1	1,556914	0,100628
**16523**	Kcnj8	potassium inwardly-rectifying channel, subfamily J, member 8	1,344428	0,113092
**16803**	Lbp	lipopolysaccharide binding protein	1,38398	0,11607
**16365**	Irg1	immunoresponsive gene 1	2,550869	0,119346
**66107**	Wfdc21	WAP four-disulfide core domain 21	1,877678	0,122404
**16193**	Il6	interleukin 6	2,0147	0,128143
**18127**	Nos3	nitric oxide synthase 3, endothelial cell	1,157462	0,145721
**11491**	Adam17	a disintegrin and metallopeptidase domain 17	1,058605	0,147737
**107765**	Ankrd1	ankyrin repeat domain 1 (cardiac muscle)	1,43967	0,162855
**18173**	Slc11a1	solute carrier family 11 (proton-coupled divalent metal ion transporters), member 1	1,302219	0,172141
**17970**	Ncf2	neutrophil cytosolic factor 2	1,202668	0,179149
**21926**	Tnf	tumor necrosis factor	1,355574	0,186093
**73914**	Irak3	interleukin-1 receptor-associated kinase 3	1,283784	0,196967
**15978**	Ifng	interferon gamma	1,466395	0,201587
**626578**	Gbp10	guanylate-binding protein 10	1,376672	0,205433
**19204**	Ptafr	platelet-activating factor receptor	1,346787	0,224385

**17874**	Myd88	myeloid differentiation primary response gene 88	1,157162	0,226358
**16162**	Il12rb2	interleukin 12 receptor, beta 2	1,304235	0,231837
**246256**	Fcgr4	Fc receptor, IgG, low affinity IV	1,47606	0,252204
**414084**	Tnip3	TNFAIP3 interacting protein 3	1,125609	0,256527
**20851**	Stat5b	signal transducer and activator of transcription 5B	1,097249	0,269314
**18035**	Nfkbia	nuclear factor of kappa light polypeptide gene enhancer in B cells inhibitor, alpha	1,105807	0,273375
**50701**	Elane	elastase, neutrophil expressed	1,265409	0,330292
**21803**	Tgfb1	transforming growth factor, beta 1	1,103445	0,353336
**20846**	Stat1	signal transducer and activator of transcription 1	1,200634	0,360058
**18126**	Nos2	nitric oxide synthase 2, inducible	1,339133	0,445759
**257632**	Nod2	nucleotide-binding oligomerization domain containing 2	0,952542	0,517038
**257632**	Nod2	nucleotide-binding oligomerization domain containing 2	1,07771	0,587887

Table S3.4: List of genes annotated with the term: GO:0032496: Response to lipopolysaccharide

Method: The 1200 PCA loadings having the lowest values (negative direction) for the second principal component of the PCA of inflamed samples were analyzed for overrepresentation of GO terms using the pcaGoPromoter package (HANSEN, M., GERDS, T. A., NIELSEN, O. H., SEIDELIN, J. B., TROELSEN, J. T. & OLSEN, J. 2012. pcaGoPromoter--an R package for biological and regulatory interpretation of principal components in genome-wide gene expression data. *PLoS One,* 7**,** e32394.)

Table S3.5

Entrez ID	Symbol	Gene Name	Fold difference KO/WT	p-val
**17537**	Meis3	Meis homeobox 3	1,199326588	3,91E-05
**13491**	Drd4	dopamine receptor D4	1,131504587	0,000468
**211323**	Nrg1	neuregulin 1	1,566098437	0,000657
**11486**	Ada	adenosine deaminase	1,30516722	0,001123
**15368**	Hmox1	heme oxygenase (decycling) 1	1,669209815	0,00466
**117149**	Tirap	toll-interleukin 1 receptor (TIR) domain-containing adaptor protein	1,199439967	0,005892
**12370**	Casp8	caspase 8	1,165535051	0,01281
**16416**	Itgb3	integrin beta 3	1,169431852	0,012896
**20361**	Sema7a	sema domain, immunoglobulin domain (Ig), and GPI membrane anchor, (semaphorin) 7A	1,6833067	0,012952
**12273**	C5ar1	complement component 5a receptor 1	1,426272406	0,015799
**21825**	Thbs1	thrombospondin 1	1,437723299	0,016175
**21817**	Tgm2	transglutaminase 2, C polypeptide	1,638305196	0,018782
**19401**	Rara	retinoic acid receptor, alpha	1,128442775	0,019123
**243983**	Zdhhc13	zinc finger, DHHC domain containing 13	1,203065004	0,019212
**12825**	Col3a1	collagen, type III, alpha 1	1,214165861	0,020336
**56532**	Ripk3	receptor-interacting serine-threonine kinase 3	1,450700372	0,022462
**19367**	Rad9a	RAD9 homolog A	1,106140068	0,026313
**16010**	Igfbp4	insulin-like growth factor binding protein 4	1,319097188	0,026372
**16852**	Lgals1	lectin, galactose binding, soluble 1	1,10529526	0,026945
**12985**	Csf3	colony stimulating factor 3 (granulocyte)	1,377737877	0,032847
**18413**	Osm	oncostatin M	1,839644436	0,033488
**59126**	Nek6	NIMA (never in mitosis gene a)-related expressed kinase 6	1,169848751	0,036665
**73230**	Bmper	BMP-binding endothelial regulator	1,293751054	0,043987
**15945**	Cxcl10	chemokine (C-X-C motif) ligand 10	2,44272561	0,044804
**16156**	Il11	interleukin 11	2,341732677	0,050261
**16011**	Igfbp5	insulin-like growth factor binding protein 5	1,373206213	0,050466
**16000**	Igf1	insulin-like growth factor 1	1,283093774	0,057446
**19303**	Pxn	paxillin	1,09387877	0,060421

**13649**	Egfr	epidermal growth factor receptor	1,14927553	0,063258
**69097**	Trim15	tripartite motif-containing 15	1,671154145	0,063874
**19697**	Rela	v-rel reticuloendotheliosis viral oncogene homolog A (avian)	1,10120395	0,068876
**64654**	Fgf23	fibroblast growth factor 23	1,672016669	0,083493
**16176**	Il1b	interleukin 1 beta	1,905150026	0,084803
**384009**	Glipr2	GLI pathogenesis-related 2	1,347589813	0,085161
**16175**	Il1a	interleukin 1 alpha	2,585228093	0,09377
**17319**	Mif	macrophage migration inhibitory factor	1,149761944	0,094491
**12654**	Chil1	chitinase-like 1	1,764722082	0,097187
**20303**	Ccl4	chemokine (C-C motif) ligand 4	1,806492031	0,098503
**20344**	Selp	selectin, platelet	1,624602838	0,103011
**56620**	Clec4n	C-type lectin domain family 4, member n	1,322179181	0,106317
**20202**	S100a9	S100 calcium binding protein A9 (calgranulin B)	1,858873089	0,115158
**14058**	F10	coagulation factor X	1,723810162	0,117468
**30937**	Lmcd1	LIM and cysteine-rich domains 1	1,308860958	0,118474
**20201**	S100a8	S100 calcium binding protein A8 (calgranulin A)	2,185588152	0,119307
**16193**	Il6	interleukin 6	2,014699865	0,128143
**72446**	Prr5l	proline rich 5 like	1,137970847	0,135018
**72287**	Plekhf1	pleckstrin homology domain containing, family F (with FYVE domain) member 1	1,12711329	0,136797
**22038**	Plscr1	phospholipid scramblase 1	1,132188148	0,14574
**66208**	Nenf	neuron derived neurotrophic factor	1,106770595	0,151392
**21832**	Thpo	thrombopoietin	1,156927997	0,155142
**107765**	Ankrd1	ankyrin repeat domain 1 (cardiac muscle)	1,439670308	0,162855
**54711**	Plagl2	pleiomorphic adenoma gene-like 2	1,080220685	0,175801
**15894**	Icam1	intercellular adhesion molecule 1	1,284343705	0,179943
**12768**	Ccr1	chemokine (C-C motif) receptor 1	1,452901228	0,183597
**211323**	Nrg1	neuregulin 1	1,072811696	0,18604
**21926**	Tnf	tumor necrosis factor	1,355574202	0,186093
**12981**	Csf2	colony stimulating factor 2 (granulocyte-macrophage)	1,097605017	0,194394
**15978**	Ifng	interferon gamma	1,466395008	0,201587
**12978**	Csf1r	colony stimulating factor 1 receptor	1,187319041	0,2133
**17874**	Myd88	myeloid differentiation primary response gene 88	1,157162361	0,226358
**14165**	Fgf10	fibroblast growth factor 10	1,17466061	0,244942

**233046**	Rasgrp4	RAS guanyl releasing protein 4	1,282928285	0,254291
**332579**	Card9	caspase recruitment domain family, member 9	1,124675567	0,256341
**14173**	Fgf2	fibroblast growth factor 2	1,207806404	0,266651
**71920**	Epgn	epithelial mitogen	1,310457831	0,272115
**14462**	Gata3	GATA binding protein 3	1,104580722	0,272671
**22339**	Vegfa	vascular endothelial growth factor A	1,197489798	0,27598
**104709**	Pik3r6	phosphoinositide-3-kinase, regulatory subunit 6	1,205384175	0,311496
**14191**	Fgr	Gardner-Rasheed feline sarcoma viral (Fgr) oncogene homolog	1,303455198	0,328007
**21943**	Tnfsf11	tumor necrosis factor (ligand) superfamily, member 11	1,172480753	0,347524
**21803**	Tgfb1	transforming growth factor, beta 1	1,103444815	0,353336
**15163**	Hcls1	hematopoietic cell specific Lyn substrate 1	1,180537411	0,407592
**78781**	Zc3hav1	zinc finger CCCH type, antiviral 1	1,032358771	0,424353
**66208**	Nenf	neuron derived neurotrophic factor	1,033584007	0,449517
**257632**	Nod2	nucleotide-binding oligomerization domain containing 2	0,952541955	0,517038
**257632**	Nod2	nucleotide-binding oligomerization domain containing 2	1,077709849	0,587887
**216869**	Arrb2	arrestin, beta 2	1,044921002	0,713337

Table S3.5: List of genes annotated with the term: GO:1902533: Positive regulation of intracellular signal transduction

Method: The 1200 PCA loadings having the lowest values (negative direction) for the second principal component of the PCA of inflamed samples were analyzed for overrepresentation of GO terms using the pcaGoPromoter package (HANSEN, M., GERDS, T. A., NIELSEN, O. H., SEIDELIN, J. B., TROELSEN, J. T. & OLSEN, J. 2012. pcaGoPromoter--an R package for biological and regulatory interpretation of principal components in genome-wide gene expression data. *PLoS One,* 7**,** e32394.)

Table S3.6

Entrez ID	Symbol	Gene Name	Fold difference KO/WT	p-val
**17395**	Mmp9	matrix metallopeptidase 9	2,107634246	0,003869
**15368**	Hmox1	heme oxygenase (decycling) 1	1,669209815	0,00466
**12827**	Col4a2	collagen, type IV, alpha 2	1,228568202	0,005979
**16416**	Itgb3	integrin beta 3	1,169431852	0,012896
**21825**	Thbs1	thrombospondin 1	1,437723299	0,016175
**105450**	Mmrn2	multimerin 2	1,216618268	0,018448
**69219**	Ddah1	dimethylarginine dimethylaminohydrolase 1	1,338569535	0,025043
**238328**	Vash1	vasohibin 1	1,115733852	0,027106
**19225**	Ptgs2	prostaglandin-endoperoxide synthase 2	2,543337484	0,03825
**20692**	Sparc	secreted acidic cysteine rich glycoprotein	1,124104477	0,038777
**11541**	Adora2b	adenosine A2b receptor	1,275129868	0,042341
**15945**	Cxcl10	chemokine (C-X-C motif) ligand 10	2,44272561	0,044804
**23892**	Grem1	gremlin 1	1,398133558	0,045941
**11504**	Adamts1	a disintegrin-like and metallopeptidase (reprolysin type) with thrombospondin type 1 motif, 1	1,37689439	0,053617
**76905**	Lrg1	leucine-rich alpha-2-glycoprotein 1	1,56334384	0,078024
**16176**	Il1b	interleukin 1 beta	1,905150026	0,084803
**13805**	Eng	endoglin	1,213596664	0,086823
**11535**	Adm	adrenomedullin	1,589629927	0,091321
**16175**	Il1a	interleukin 1 alpha	2,585228093	0,09377
**20692**	Sparc	secreted acidic cysteine rich glycoprotein	1,224756316	0,095072
**12654**	Chil1	chitinase-like 1	1,764722082	0,097187
**20296**	Ccl2	chemokine (C-C motif) ligand 2	1,864354159	0,099166
**18787**	Serpine1	serine (or cysteine) peptidase inhibitor, clade E, member 1	1,556913819	0,100628
**257630**	Il17f	interleukin 17F	1,246722271	0,102746
**22375**	Wars	tryptophanyl-tRNA synthetase	1,183673871	0,11068
**12765**	Cxcr2	chemokine (C-X-C motif) receptor 2	1,63756528	0,140666
**18127**	Nos3	nitric oxide synthase 3, endothelial cell	1,157461651	0,145721
**12765**	Cxcr2	chemokine (C-X-C motif) receptor 2	1,483750117	0,148695

**12796**	Camp	cathelicidin antimicrobial peptide	1,500774312	0,211452
**12774**	Ccr5	chemokine (C-C motif) receptor 5	1,384260071	0,241965
**14173**	Fgf2	fibroblast growth factor 2	1,207806404	0,266651
**58861**	Cysltr1	cysteinyl leukotriene receptor 1	1,165812512	0,273729
**22339**	Vegfa	vascular endothelial growth factor A	1,197489798	0,27598
**240725**	Sulf1	sulfatase 1	1,208398169	0,277186
**12772**	Ccr2	chemokine (C-C motif) receptor 2	1,362893367	0,27726
**104709**	Pik3r6	phosphoinositide-3-kinase, regulatory subunit 6	1,205384175	0,311496
**16414**	Itgb2	integrin beta 2	1,219791673	0,322302
**20846**	Stat1	signal transducer and activator of transcription 1	1,200633731	0,360058
**16414**	Itgb2	integrin beta 2	1,090503511	0,523318
**11541**	Adora2b	adenosine A2b receptor	1,030537746	0,63402

Table S3.6: List of genes annotated with the term: GO:0045765: Regulation of angiogenesis

Method: The 1200 PCA loadings having the lowest values (negative direction) for the second principal component of the PCA of inflamed samples were analyzed for overrepresentation of GO terms using the pcaGoPromoter package (HANSEN, M., GERDS, T. A., NIELSEN, O. H., SEIDELIN, J. B., TROELSEN, J. T. & OLSEN, J. 2012. pcaGoPromoter--an R package for biological and regulatory interpretation of principal components in genome-wide gene expression data. *PLoS One,* 7**,** e32394.)

Table S3.7

Entrez ID	Symbol	Gene Name	Fold difference KO/WT	p-val
**17537**	Meis3	Meis homeobox 3	1,199326588	3,91E-05
**73699**	Ppp2r1b	protein phosphatase 2, regulatory subunit A, beta	1,374029959	0,000471
**11486**	Ada	adenosine deaminase	1,30516722	0,001123
**12424**	Cck	cholecystokinin	1,679692197	0,001615
**110886**	Gabra5	gamma-aminobutyric acid (GABA) A receptor, subunit alpha 5	1,182016303	0,002404
**27053**	Asns	asparagine synthetase	1,380942089	0,00264
**17395**	Mmp9	matrix metallopeptidase 9	2,107634246	0,003869
**15368**	Hmox1	heme oxygenase (decycling) 1	1,669209815	0,00466
**13063**	Cycs	cytochrome c, somatic	1,154488776	0,004924
**67603**	Dusp6	dual specificity phosphatase 6	1,490947538	0,005299
**74041**	Ddias	DNA damage-induced apoptosis suppressor	1,364031646	0,00764
**21857**	Timp1	tissue inhibitor of metalloproteinase 1	1,915117509	0,009413
**12370**	Casp8	caspase 8	1,165535051	0,01281
**13063**	Cycs	cytochrome c, somatic	1,230587834	0,014252
**13063**	Cycs	cytochrome c, somatic	1,230587834	0,014252
**216150**	Cdc34	cell division cycle 34	1,216534479	0,015146
**12273**	C5ar1	complement component 5a receptor 1	1,426272406	0,015799
**19713**	Ret	ret proto-oncogene	1,457300898	0,016129
**21825**	Thbs1	thrombospondin 1	1,437723299	0,016175
**18103**	Nme2	NME/NM23 nucleoside diphosphate kinase 2	1,161391226	0,016867
**12018**	Bak1	BCL2-antagonist/killer 1	1,234227586	0,018541
**21817**	Tgm2	transglutaminase 2, C polypeptide	1,638305196	0,018782
**19401**	Rara	retinoic acid receptor, alpha	1,128442775	0,019123
**21933**	Tnfrsf10b	tumor necrosis factor receptor superfamily, member 10b	1,437228857	0,021811
**56532**	Ripk3	receptor-interacting serine-threonine kinase 3	1,450700372	0,022462
**13063**	Cycs	cytochrome c, somatic	1,18466242	0,024903
**268973**	Nlrc4	NLR family, CARD domain containing 4	1,231243659	0,026239
**19367**	Rad9a	RAD9 homolog A	1,106140068	0,026313
**16323**	Inhba	inhibin beta-A	1,696159573	0,026631

**12051**	Bcl3	B cell leukemia/lymphoma 3	1,297998438	0,026953
**14283**	Fosl1	fos-like antigen 1	1,57055952	0,027673
**18413**	Osm	oncostatin M	1,839644436	0,033488
**19225**	Ptgs2	prostaglandin-endoperoxide synthase 2	2,543337484	0,03825
**13063**	Cycs	cytochrome c, somatic	1,11367673	0,03833
**20656**	Sod2	superoxide dismutase 2, mitochondrial	1,166716868	0,04205
**64136**	Sdf2l1	stromal cell-derived factor 2-like 1	1,247183848	0,042527
**20845**	Star	steroidogenic acute regulatory protein	1,098991401	0,044186
**57875**	Angptl4	angiopoietin-like 4	1,717352117	0,044406
**15251**	Hif1a	hypoxia inducible factor 1, alpha subunit	1,192255184	0,04658
**18793**	Plaur	plasminogen activator, urokinase receptor	1,792923445	0,049392
**50501**	Prok2	prokineticin 2	3,260128099	0,050298
**20378**	Frzb	frizzled-related protein	1,287729133	0,051764
**56369**	Apip	APAF1 interacting protein	1,144829157	0,051965
**12064**	Bdnf	brain derived neurotrophic factor	1,099755919	0,054715
**12608**	Cebpb	CCAAT/enhancer binding protein (C/EBP), beta	1,406471906	0,056195
**16000**	Igf1	insulin-like growth factor 1	1,283093774	0,057446
**13649**	Egfr	epidermal growth factor receptor	1,14927553	0,063258
**14373**	G0s2	G0/G1 switch gene 2	1,145498031	0,065804
**19697**	Rela	v-rel reticuloendotheliosis viral oncogene homolog A (avian)	1,10120395	0,068876
**68497**	Arel1	apoptosis resistant E3 ubiquitin protein ligase 1	1,071712913	0,076271
**17390**	Mmp2	matrix metallopeptidase 2	1,127686341	0,080369
**16176**	Il1b	interleukin 1 beta	1,905150026	0,084803
**216799**	Nlrp3	NLR family, pyrin domain containing 3	1,919111518	0,08831
**20302**	Ccl3	chemokine (C-C motif) ligand 3	2,70627181	0,090362
**11535**	Adm	adrenomedullin	1,589629927	0,091321
**17319**	Mif	macrophage migration inhibitory factor	1,149761944	0,094491
**18787**	Serpine1	serine (or cysteine) peptidase inhibitor, clade E, member 1	1,556913819	0,100628
**18712**	Pim1	proviral integration site 1	1,372822858	0,10249
**14419**	Gal	galanin	1,389857646	0,103806
**20202**	S100a9	S100 calcium binding protein A9 (calgranulin B)	1,858873089	0,115158
**14373**	G0s2	G0/G1 switch gene 2	1,316090991	0,115814
**107503**	Atf5	activating transcription factor 5	1,098877053	0,115909

**54683**	Prdx5	peroxiredoxin 5	1,157131171	0,116257
**20201**	S100a8	S100 calcium binding protein A8 (calgranulin A)	2,185588152	0,119307
**18103**	Nme2	NME/NM23 nucleoside diphosphate kinase 2	1,15362961	0,121708
**19252**	Dusp1	dual specificity phosphatase 1	1,257841984	0,12324
**16193**	Il6	interleukin 6	2,014699865	0,128143
**72287**	Plekhf1	pleckstrin homology domain containing, family F (with FYVE domain) member 1	1,12711329	0,136797
**19252**	Dusp1	dual specificity phosphatase 1	1,236245786	0,138226
**12661**	Chl1	cell adhesion molecule with homology to L1CAM	1,471456546	0,140029
**12765**	Cxcr2	chemokine (C-X-C motif) receptor 2	1,63756528	0,140666
**11911**	Atf4	activating transcription factor 4	1,06207688	0,141146
**14127**	Fcer1g	Fc receptor, IgE, high affinity I, gamma polypeptide	1,330685841	0,14568
**18127**	Nos3	nitric oxide synthase 3, endothelial cell	1,157461651	0,145721
**22038**	Plscr1	phospholipid scramblase 1	1,132188148	0,14574
**20379**	Sfrp4	secreted frizzled-related protein 4	1,231803969	0,146716
**11491**	Adam17	a disintegrin and metallopeptidase domain 17	1,058605094	0,147737
**12765**	Cxcr2	chemokine (C-X-C motif) receptor 2	1,483750117	0,148695
**11911**	Atf4	activating transcription factor 4	1,081575923	0,156514
**107765**	Ankrd1	ankyrin repeat domain 1 (cardiac muscle)	1,439670308	0,162855
**13166**	Dbh	dopamine beta hydroxylase	1,287502229	0,163012
**80885**	Hcar2	hydroxycarboxylic acid receptor 2	1,663089892	0,170479
**54711**	Plagl2	pleiomorphic adenoma gene-like 2	1,080220685	0,175801
**50930**	Tnfsf14	tumor necrosis factor (ligand) superfamily, member 14	1,220854404	0,175822
**20723**	Serpinb9	serine (or cysteine) peptidase inhibitor, clade B, member 9	1,192457921	0,176747
**17970**	Ncf2	neutrophil cytosolic factor 2	1,202667924	0,179149
**15894**	Icam1	intercellular adhesion molecule 1	1,284343705	0,179943
**20343**	Sell	selectin, lymphocyte	1,503068041	0,181181
**18712**	Pim1	proviral integration site 1	1,127744372	0,182157
**21926**	Tnf	tumor necrosis factor	1,355574202	0,186093
**12981**	Csf2	colony stimulating factor 2 (granulocyte-macrophage)	1,097605017	0,194394
**73914**	Irak3	interleukin-1 receptor-associated kinase 3	1,283784357	0,196967
**216150**	Cdc34	cell division cycle 34	1,102342759	0,197228
**15978**	Ifng	interferon gamma	1,466395008	0,201587

**58231**	Stk4	serine/threonine kinase 4	1,0528485	0,207362
**22353**	Vip	vasoactive intestinal polypeptide	1,280018788	0,223114
**68655**	Fndc1	fibronectin type III domain containing 1	1,097383815	0,226659
**12257**	Tspo	translocator protein	1,125934936	0,237956
**12774**	Ccr5	chemokine (C-C motif) receptor 5	1,384260071	0,241965
**14165**	Fgf10	fibroblast growth factor 10	1,17466061	0,244942
**13032**	Ctsc	cathepsin C	1,11414773	0,263953
**20851**	Stat5b	signal transducer and activator of transcription 5B	1,097248895	0,269314
**14462**	Gata3	GATA binding protein 3	1,104580722	0,272671
**22339**	Vegfa	vascular endothelial growth factor A	1,197489798	0,27598
**18124**	Nr4a3	nuclear receptor subfamily 4, group A, member 3	1,163314674	0,277728
**23845**	Clec5a	C-type lectin domain family 5, member a	1,324283295	0,291246
**14102**	Fas	Fas (TNF receptor superfamily member 6)	1,173604831	0,309788
**11911**	Atf4	activating transcription factor 4	1,052426373	0,326959
**15162**	Hck	hemopoietic cell kinase	1,201206064	0,339102
**12477**	Ctla4	cytotoxic T-lymphocyte-associated protein 4	1,177467407	0,345599
**21803**	Tgfb1	transforming growth factor, beta 1	1,103444815	0,353336
**20846**	Stat1	signal transducer and activator of transcription 1	1,200633731	0,360058
**66168**	Grina	glutamate receptor, ionotropic, N-methyl D-aspartate-associated protein 1 (glutamate binding)	1,127613982	0,378489
**15163**	Hcls1	hematopoietic cell specific Lyn substrate 1	1,180537411	0,407592
**15251**	Hif1a	hypoxia inducible factor 1, alpha subunit	1,072318289	0,484426
**257632**	Nod2	nucleotide-binding oligomerization domain containing 2	0,952541955	0,517038
**257632**	Nod2	nucleotide-binding oligomerization domain containing 2	1,077709849	0,587887
**216869**	Arrb2	arrestin, beta 2	1,044921002	0,713337
**67603**	Dusp6	dual specificity phosphatase 6	1,029408062	0,746953
**15251**	Hif1a	hypoxia inducible factor 1, alpha subunit	1,01026005	0,947384

Table S3.7: List of genes annotated with the term: GO:0042981: Regulation of apoptotic process

Method: The 1200 PCA loadings having the lowest values (negative direction) for the second principal component of the PCA of inflamed samples were analyzed for overrepresentation of GO terms using the pcaGoPromoter package (HANSEN, M., GERDS, T. A., NIELSEN, O. H., SEIDELIN, J. B., TROELSEN, J. T. & OLSEN, J. 2012. pcaGoPromoter--an R package for biological and regulatory interpretation of principal components in genome-wide gene expression data. *PLoS One,* 7**,** e32394.)

Table S4.1

		PWM	Length	Factor name	P-value
**1**	10132	PB0010	14	Egr1_1	7,73E-06
**2**	9286	MA0058	10	MAX	3,06E-04
**3**	9232	MA0004	6	Arnt	4,57E-04
**4**	9321	MA0093	7	USF1	4,57E-04
**5**	9326	MA0098	6	ETS1	4,57E-04
**6**	9332	MA0104	6	Mycn	4,57E-04
**7**	9400	MA0162	11	Egr1	4,57E-04
**8**	9231	MA0003	9	TFAP2A	6,99E-04
**9**	10174	PB0052	16	Plagl1_1	9,63E-04
**10**	9380	MA0039	10	Klf4	1,18E-03
**11**	10224	PB0102	15	Zic2_1	1,28E-03
**12**	9503	MA0259	8	HIF1A::ARNT	1,45E-03
**13**	9384	MA0104	10	Mycn	2,04E-03
**14**	10131	PB0009	15	E2F3_1	2,54E-03
**15**	10130	PB0008	15	E2F2_1	2,93E-03
**16**	10225	PB0103	15	Zic3_1	2,93E-03
**17**	9295	MA0067	8	Pax2	2,96E-03
**18**	10217	PB0095	16	Zfp161_1	3,48E-03
**19**	10161	PB0039	16	Klf7_1	4,47E-03
**20**	9288	MA0060	16	NFYA	6,01E-03
**21**	9360	MA0131	10	MIZF	6,84E-03
**22**	9406	MA0079	10	SP1	7,08E-03
**23**	9308	MA0080	6	SPI1	7,26E-03
**24**	10273	PB0151	15	Myf6_2	7,26E-03
**25**	10321	PB0199	14	Zfp161_2	7,26E-03
**26**	10271	PB0149	16	Myb_2	9,10E-03
**27**	10328	PB0206	15	Zic2_2	9,10E-03
**28**	10210	PB0088	15	Tcfap2e_1	1,04E-02
**29**	10286	PB0164	17	Smad3_2	1,12E-02
**30**	9375	MA0147	10	Myc	1,42E-02
**31**	9331	MA0103	6	ZEB1	1,77E-02
**32**	9283	MA0055	12	Myf	2,01E-02

**33**	10239	PB0117	16	Eomes_2	2,01E-02
**34**	10275	PB0153	16	Nr2f2_2	2,01E-02
**35**	9374	MA0146	14	Zfx	2,50E-02
**36**	10209	PB0087	15	Tcfap2c_1	2,99E-02
**37**	10302	PB0180	15	Sp4_2	2,99E-02
**38**	9341	MA0112	18	ESR1	3,15E-02
**39**	9401	MA0163	14	PLAG1	3,15E-02
**40**	10223	PB0101	14	Zic1_1	3,52E-02
**41**	10129	PB0007	22	Bhlhb2_1	3,97E-02
**42**	10312	PB0190	15	Tcfap2b_2	3,97E-02
**43**	10327	PB0205	15	Zic1_2	3,97E-02
**44**	10156	PB0034	15	Irf4_1	4,38E-02
**45**	10165	PB0043	16	Max_1	4,38E-02
**46**	10198	PB0076	17	Sp4_1	4,38E-02

Table S4.1: Overrepresented inflamed PC2 positive

Table S4.2

		PWM	Length	Name	P-value
**1**	9289	MA0061	10	NF-kappaB	0,00269
**2**	9333	MA0105	11	NFKB1	0,011729
**3**	9278	MA0050	12	IRF1	0,015693
**4**	9279	MA0051	18	IRF2	0,015693
**5**	9382	MA0002	11	RUNX1	0,015693
**6**	10180	PB0058	14	Sfpi1_1	0,015693
**7**	10494	PH0164	17	Six4	0,015693
**8**	10278	PB0156	17	Plagl1_2	0,017356
**9**	9335	MA0107	10	RELA	0,020635
**10**	10125	PB0003	17	Ascl2_1	0,029336
**11**	10155	PB0033	14	Irf3_1	0,029336
**12**	10207	PB0085	15	Tcfap2a_1	0,029336
**13**	9257	MA0029	14	Evi1	0,038333
**14**	9284	MA0056	6	MZF1_1-4	0,038333
**15**	10212	PB0090	17	Zbtb12_1	0,038333
**16**	9502	MA0258	18	ESR2	0,041977
**17**	10323	PB0201	17	Zfp281_2	0,041977
**18**	9230	MA0002	11	RUNX1	0,04271
**19**	9318	MA0090	12	TEAD1	0,044985
**20**	9373	MA0145	14	Tcfcp2l1	0,044985
**21**	10146	PB0024	16	Gcm1_1	0,044985
**22**	9309	MA0081	7	SPIB	0,048046
**23**	9329	MA0101	10	REL	0,048046
**24**	9365	MA0137	14	STAT1	0,048046
**25**	10311	PB0189	14	Tcfap2a_2	0,048046
**26**	10312	PB0190	15	Tcfap2b_2	0,048046

Table S4.2: Overrepresented inflamed PC2 negative
